# CTRP4/interleukin-6 receptor signaling ameliorates autoimmune encephalomyelitis by suppressing Th17 cell differentiation

**DOI:** 10.1172/JCI168384

**Published:** 2023-11-28

**Authors:** Lulu Cao, Jinhai Deng, Wei Chen, Minwei He, Ning Zhao, He Huang, Lu Ling, Qi Li, Xiaoxin Zhu, Lu Wang

**Affiliations:** 1 Department of Rheumatology and Immunology, Peking University People’s Hospital and Beijing Key Laboratory for Rheumatism Mechanism and Immune Diagnosis (BZ0135), Beijing, China.; 2 Department of Immunology, School of Basic Medical Sciences, Health Science Center, and; 3 Key Laboratory of Medical Immunology, Ministry of Health, School of Basic Medical Science, Peking University, Beijing, China.; 4 Department of Clinical Laboratory, Beijing Chaoyang Hospital, Capital Medical University, Beijing, China.; 5 Institute of Chinese Materia Medica, China Academy of Chinese Medical Sciences, Beijing, China.

**Keywords:** Autoimmunity, Cellular immune response

## Abstract

C1q/TNF-related protein 4 (CTRP4) is generally thought to be released extracellularly and plays a critical role in energy metabolism and protecting against sepsis. However, its physiological functions in autoimmune diseases have not been thoroughly explored. In this study, we demonstrate that Th17 cell–associated experimental autoimmune encephalomyelitis was greatly exacerbated in *Ctrp4^–/–^* mice compared with WT mice due to increased Th17 cell infiltration. The absence of *Ctrp4* promoted the differentiation of naive CD4^+^ T cells into Th17 cells in vitro. Mechanistically, CTRP4 interfered with the interaction between IL-6 and the IL-6 receptor (IL-6R) by directly competing to bind with IL-6R, leading to suppression of IL-6–induced activation of the STAT3 pathway. Furthermore, the administration of recombinant CTRP4 protein ameliorated disease symptoms. In conclusion, our results indicate that CTRP4, as an endogenous regulator of the IL-6 receptor–signaling pathway, may be a potential therapeutic intervention for Th17-driven autoimmune diseases.

## Abstract 2

<p>CTRP4 suppresses IL-6-induced activation of the STAT3 pathway by binding to IL-6R in Th17 cell-associated experimental autoimmune encephalomyelitis.</p>

## Graphical abstract

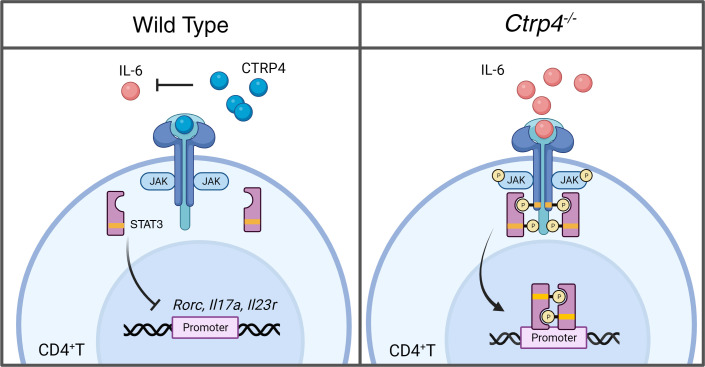

## Introduction

Multiple sclerosis (MS) is an inflammatory disorder of the CNS identified by chronic demyelination and axonal damage. Upon activation in the peripheral lymphoid organs, the autoimmune T cells enter the CNS through the blood-brain barrier and become reactivated, resulting in the enrichment of leukocytes and disseminated inflammation, demyelination, and symptoms of severe disease (1, 2). Experimental autoimmune encephalomyelitis (EAE) has been reported to be one of the most frequently used animal models of MS. The initiation of EAE is associated with peripheral priming of myelin-specific dysregulated Th1 and Th17 cells (2), which contribute to the pathogenesis of MS and highly correlate with disease severity and relapse frequency (3). Th17 cells categorized as pathogenic or nonpathogenic subtypes, depending on the cytokine milieu, display considerable plasticity (4). TGF-β and IL-6 are the main factors for driving classical Th17 cell differentiation (5), while IL-6, IL-23, and IL-1β cytokines trigger Th17 to develop pathogenic functions with tissue-destructive properties (6).

IL-6 is an integral cytokine responsible for the transcriptional programming of Th17 cells. In addition to having a key role in Th17 cell induction, IL-6 is necessary for retaining the transcriptional and functional identities of Th17 (7), which is indispensable for EAE disease progression. There appear to be multiple benefits associated with interfering with the IL-6/STAT3 signaling axis in the treatment of autoimmune diseases (8). Assembly of a complex between IL-6 and different forms of the IL-6 receptor (IL-6R) is required for the transmission of different types of IL-6 signaling, including classical signaling, trans-signaling, and transpresentation signaling. Classic signaling is triggered by the binding of IL-6 to the membrane form of IL-6R, followed by the activation of gp130 and the sequential recruitment of STAT3. Although gp130 is ubiquitously expressed, membrane IL-6R expression is restricted to limited cells. Soluble IL-6R (sIL-6R) is a critical supplement for the activation of IL-6 signaling in cells not expressing IL-6R on the surface through a process termed trans-signaling. Recently, an additional mode termed transpresentation has been identified: DC-expressed IL-6R binds to IL-6 and then forms a complex, followed by interacting with gp130-expressing T cells, leading to the differentiation of pathogenic Th17 cells.

C1q/TNF-related protein 4 (CTRP4) featuring 2 highly conserved complement C1q domains is a classical secreted protein. As a metabolic regulator, CTRP4 secreted from the brain modulates food intake and body weight (9, 10). Furthermore, its role in the CNS has been extended, and it has been found that the deletion of CTRP4 impaired hippocampal-dependent learning and memory of mice (11). Other studies have highlighted a potential role for CTRP4 in the immune system. For instance, CTRP4 inhibited the progression of colorectal cancer (12) and the absence of CTRP4 in a sepsis model was also associated with exacerbated activation of macrophages with TLR4 internalization, leading to inflammatory cytokine release (13). In addition, exome sequencing of systemic lupus erythematosus patients identified a rare mutation of CTRP4/C1QTNF4 (H198Q) that inhibited TNF-mediated NF-κB activation and cell death (14), suggesting potential role of CTRP4 in autoimmune diseases. In spite of this, there have been conflicting reports until now: the proinflammatory role of CTRP4 has been observed in cancer-related inflammation, while antiinflammatory properties have been observed in other inflammatory settings. Although nucleolin (a shuttling protein) has been identified as a cell-surface docking protein binding CTRP4 on monocytes and dead cells (15), this paradox cannot be explained. Therefore, other potential receptors may exist and need to be investigated for a deeper understanding of the precise molecular mechanisms of CTRP4 in physiological and pathological settings.

In this study, we evaluated the role of CTRP4 in Th17 cell homing, priming, and differentiation during induction and progression of EAE. Our results found that CTRP4 deficiency exacerbated symptoms in a T cell–intrinsic manner, possibly owing to its role in preferential Th17 differentiation. Mechanistically, we identified a previously unreported interaction between CTRP4 and IL-6R and subsequently inhibited IL-6/IL-6R binding, thereby suppressing STAT3 activation. Thus, our findings highlight the possible biological relevant ligand for IL-6R, which is of great importance for filling out the current knowledge of the IL-6/IL-6R/gp130 buffer system, and provide a potential inhibitor for clinical application in autoimmune diseases.

## Results

### CTRP4 deficiency impairs peripheral T cell homeostasis.

**Figure 1:**
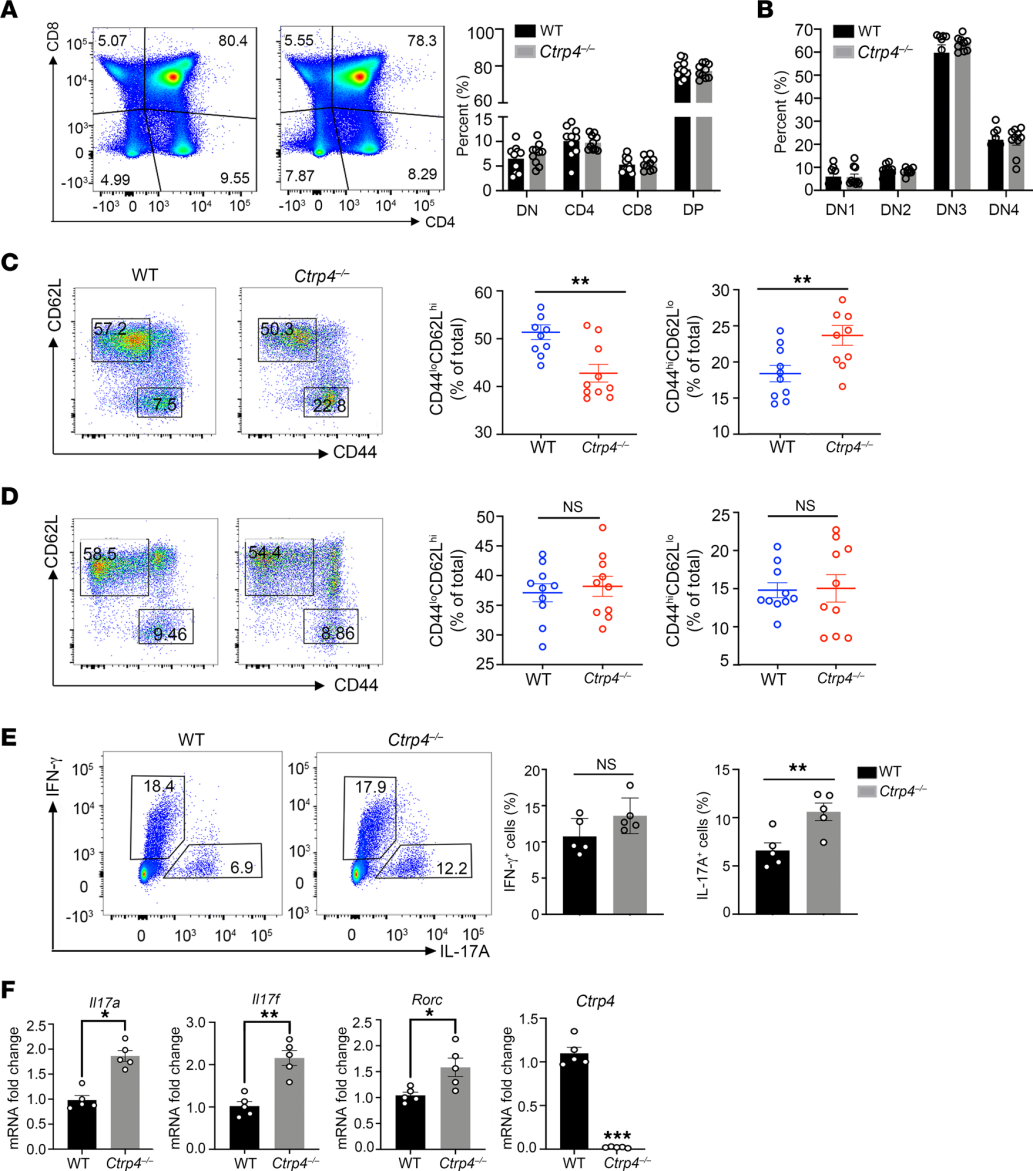
*Ctrp4* deficiency perturbs T cell homeostasis. (**A**) Surface staining of CD4 and CD8 on *Ctrp4^–/–^* or WT thymocytes. Numbers in quadrants indicate the percentages of different stage cells, including CD4^–^CD8^–^ DN, CD4^+^CD8^+^ DP, CD8^–^CD4^+^ single-positive, and CD4^–^CD8^+^ single-positive T cells (*n* = 10 animals per group from 1 representative experiment of 3). (**B**) Flow cytometry analysis of the transition between the different populations of DN T cell precursors in the thymus, which were marked by the differential expression of CD44 and CD25. DN1:CD44^+^CD25^–^, DN2:CD44^+^CD25^+^; DN3:CD44^–^CD25^+^; DN4: CD44^–^CD25^–^. (**C** and **D**) Representative plots showed the percentages of naive (CD44^lo^CD62L^hi^) and memory/effector (CD44^hi^CD62L^lo^) CD4^+^ T cells (**C**) and CD8^+^ T cells (**D**) in the spleens of *Ctrp4^–/–^* or WT mice. (**E**) Flow cytometry analyses of Th1 (IFN-γ^+^) and Th17 (IL-17A^+^) effector T cells in the spleens of *Ctrp4^–/–^* and WT mice (*n* = 5/group). Data are represented as the frequency of CD4^+^CD44^+^ cells. (**F**) Gene expression of *Il17a*, *Il17f*, *Rorc*, or *Ctrp4* mRNA in CD4^+^ T cells from *Ctrp4^–/–^* or WT mice (*n* = 5/group) were analyzed by quantitative real-time PCR. Values were normalized against *Gapdh*. Data are represented as mean ± SEM and are from 1 of 3 independent experiments with similar results. Statistical significance was determined using 2-tailed, unpaired Student’s *t* test or Mann-Whitney *U* test as appropriate after assessing for distribution. *P < 0.05; **P < 0.001.

The previous observation of preferential expression of *Ctrp4* raised the possibility that CTRP4 is functionally expressed in T cells (13). In this work, we first examined the distribution of the 4 major thymic populations and found the percentages of CD4^–^CD8^–^ double-negative (DN), CD4^+^CD8^+^ double-positive (DP), CD4^+^, and CD8^+^ single-positive (SP) thymocytes were comparable between *Ctrp4^–/–^* and control mice (Figure 1A). Furthermore, analysis of DN subsets revealed no detectable differences in the percentages of DN1, DN2, DN3, and DN4 in *Ctrp4^–/–^* mice, suggesting that CTRP4 was dispensable for T cell development in the thymus (Figure 1B). In homeostatic conditions, the peripheral T cell pool is primarily composed of naive T cells, but with increasing age, the pool remains fairly constant and begins to expand in memory-like cells characterized by CD44^hi^CD62L^lo^ markers as a result of homeostatic proliferation induced by self-peptides/MHC ligands. The increase in memory-like CD4^+^ T cells in *Ctrp4^–/–^* mice was observed along with a compensatory reduction in naive CD4^+^ T cells (Figure 1C), while the proportion of memory-like CD8^+^ T cells showed no discrepancy in the spleens (Figure 1D).

Subsequently, we examined the effector T cell subsets in the periphery. Among the CD44^hi^ memory T cell population in the spleen, Th17 cells were increased in the *Ctrp4^–/–^* mice (Figure 1E), while the percentages of Th1 and Th2 cells remained unchanged compared with the counterparts in the WT mice (Figure 1E and Supplemental Figure 1A; supplemental material available online with this article; https://doi.org/10.1172/JCI168384DS1). Moreover, the frequency of Tregs was similar between WT and *Ctrp4^–/–^* mice (Supplemental Figure 1B). To further confirm which CD4^+^ subset was more closely involved, we measured the expression levels of various Th cell signature genes. The results indicated that the levels of Th17 cell lineage–specific genes (*Il17a*, *Il17f*, and *Rorc*) were upregulated in *Ctrp4^–/–^* mice (Figure 1F), whereas the levels of genes (*Tbx21*, *Gata3*, and *Foxp3*) remained unaltered (Supplemental Figure 1C). Of note, the mRNA levels of *Ctrp4* in CD4^+^ T cells activated with anti-CD3 and anti-CD28 antibodies were not significantly upregulated relative to those of naive CD4^+^ T cells. During TCR activation in a particular cytokine milieu, naive CD4^+^ T cells could differentiate into a variety of different lineages of Th cells. The results showed that the expression of *Ctrp4* was limited in Th1 and Tregs, but significantly increased in both Th17 and Th2 cells (Supplemental Figure 1D). In line with the change of *Ctrp4* transcriptional levels, increased protein levels in Th17 cell supernatants were observed (Supplemental Figure 1E). Thus, we inferred that CTRP4 was involved in regulating peripheral T cell homeostasis, particularly that of Th17 cells of effector CD4^+^ T cells.

### CTRP4 production by CD4^+^ T cells alleviates EAE symptoms.

**Figure 2:**
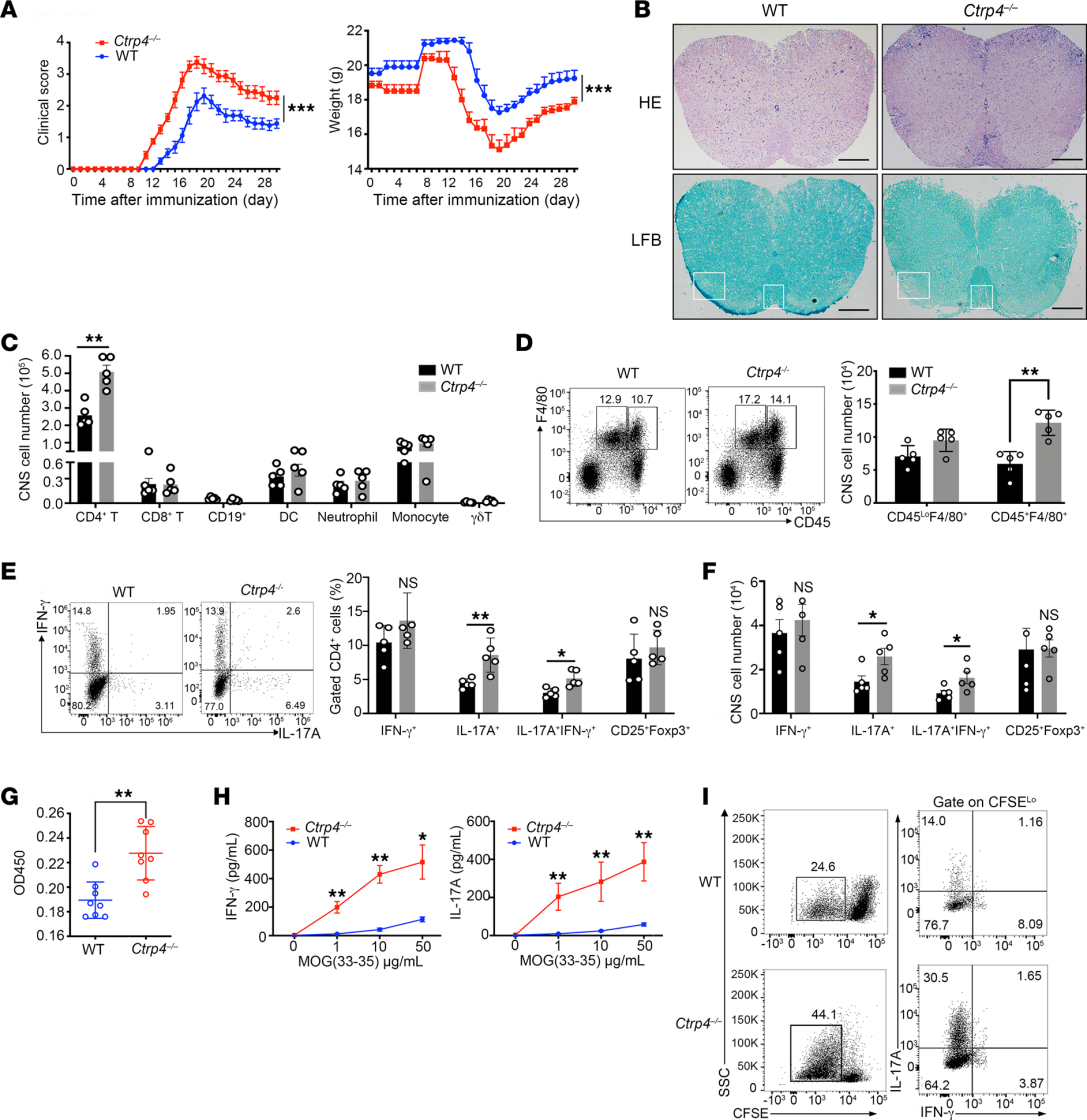
*Ctrp4* deficiency exacerbates EAE progression with increased infiltration of CD4^+^ T cells in the CNS. (**A**) After immunization with MOG peptide, the progression of disease was monitored. Clinical EAE scores and body weights of *Ctrp4^–/–^*(*n* = 10) or WT (*n* = 10) mice following disease induction are shown. Statistical significance was determined using 2-way repeated measures ANOVA. (**B**) Spinal cord sections were stained with H&E or Luxol fast blue (LFB). Histological images are representative of 3 mice in each group. Scale bars: 200 μm. (**C** and **D**) Single cells were isolated from the CNS on day 18 after EAE induction and stained with the indicated cell type–specific markers. Summary bar graph shows absolute numbers of CNS-infiltrating immune cells in control and *Ctrp4^–/–^* mice (**C**). Representative flow cytometry plots of macrophage (CD45^hi^F4/80^+^) and microglia (CD45^lo^F4/80^+^) infiltrated in CNS are shown (**D**). (**E** and **F**) Representative flow cytometry plots showed percentages of IFN-γ^+^CD4^+^, IL-17A^+^CD4^+^, and IFN-γ^+^IL-17A^+^CD4^+^ in CNS of WT and *Ctrp4^–/–^* mice (*n* = 5 mice per group) on day 18 after EAE induction. Quantified percentages (**E**) and absolute cell numbers (**F**) are shown. (**G** and **H**) Recall response of antigen-specific T cells from the dLNs of WT and *Ctrp4^–/–^* mice on day 9 after EAE induction. CD4^+^ T cells were expanded with irradiated autologous-presenting cells plus 10 μg/mL (**G**) or the indicated concentration (**H**) of MOG peptide for 72 hours and subjected to cell-proliferation assay to determine T cell recall response based on BrdU assay (**G**) and ELISA assay (**H**) to quantitate the production of IL-17A and IFN-γ. (**I**) Flow cytometric analysis of CFSE-labeled CD4^+^ T cells and quantification of intracellular cytokine staining at day 5 after in vitro coculturing with irradiated autologous-presenting cells plus 10 μg/mL MOG peptide. The percentages of IL-17^+^ and IFN-γ^+^ cells were gated on CSFE^lo^CD4^+^ T cells, and data are presented as representative flow plots. Data are represented as mean ± SEM and are from 1 of 3 independent experiments with similar results. (**C**–**H**) Statistical significance was determined using 2-tailed, unpaired Student’s *t* test or Mann-Whitney *U* test as appropriate after assessing for distribution. *P < 0.05; **P < 0.001.

To gain further insight into the pathophysiological roles of CTRP4 in T cell–mediated autoimmune disease, we studied disease progression in an EAE model to mimic human MS. After EAE induction, *Ctrp4^–/–^* mice developed disease earlier and lost more body weight, and the clinical scores gradually reached a high peak at day 17. Due to slower remission, a higher average disease score was observed in *Ctrp4^–/–^* mice (Figure 2A). Histological staining demonstrated increased immune cell infiltration and demyelination in the spinal cords of *Ctrp4^–/–^* mice (Figure 2B), suggesting a role for CTRP4 in alleviating EAE progression and occurrence.

We then examined the composition of recruited immune cells in the CNS. Compared with WT mice, in *Ctrp4^–/–^* mice, immunophenotyping combined with intracellular cytokine staining showed a higher number of CD4^+^ T cells (Figure 2C and Supplemental Figure 2A). In addition, the increased numbers of CNS-infiltrating active macrophages (CD45^+^F4/80^+^) were present in *Ctrp4^–/–^* mice during the peak phase of EAE (Figure 2D). The exacerbated disease observed in *Ctrp4^–/–^* mice was associated with a significant increase of CD4^+^IL-17A^+^ and CD4^+^IL-17A^+^ IFN-γ^+^ T cells (Figure 2, E and F). Moreover, no detectable differences were observed in the percentages of CD4^+^IFN-γ^+^ and CD4^+^CD25^+^Foxp3^+^ cells derived from WT and CTRP4-deficient mice (Figure 2, E and F). Subsequently, we investigated the abundance of CD4^+^ T cell subsets in peripheral lymphoid organs. The total number of CD4^+^ T cells was comparable in control and *Ctrp4^–/–^* mice (Supplemental Figure 2B). However, with respect to the subpopulations of CD4^+^, the numbers of IL-17A^+^ and IL-17A^+^IFN-γ^+^ CD4^+^ T cells were significantly increased in the draining lymph nodes (dLNs) and spleen of *Ctrp4^–/–^* mice (Supplemental Figure 2, C–F). Based on our findings, we inferred that increased peripheral Th17 cells were responsible for the enrichment of CNS-infiltrating CD4^+^ cells, especially Th17 cells.

To evaluate the potential of *Ctrp4* in antigen-specific expansion of CD4^+^ T cells, we accessed the recall response of MOG_35–55_-specific T cells isolated at the early effector phase of EAE progression. Upon restimulation with MOG peptides, the antigen-specific T cells from *Ctrp4*-deficient mice substantially enhanced proliferative activity (Figure 2G) and remarkably produced more IL-17A and IFN-γ, which positively correlated with clinical symptoms (Figure 2H). By gating CFSE^lo^CD4^+^ T cells, MOG_35–55_-peptide–specific Th17 cells were observed in CTRP4-deficient mice (Figure 2I).

**Figure 3:**
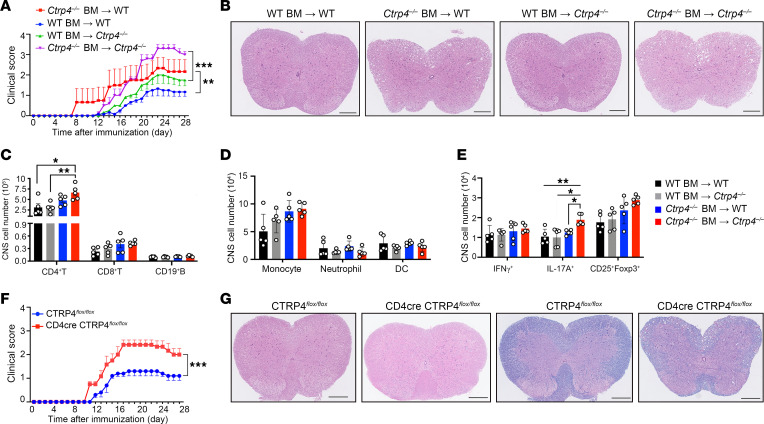
The protective function of CTRP4 is T cell intrinsic for IL-6 signaling. (**A**) Mean clinical scores of chimeric mice generated by (i) transfer of WT bone marrow cells into irradiated WT and *Ctrp4^–/–^* recipient mice or (ii) transfer of *Ctrp4^–/–^* bone marrow cells into irradiated WT and *Ctrp4^–/–^* recipient mice (each *n* = 6–8) following MOG_35–55_ immunization. Statistical significance was determined using 2-way repeated measures ANOVA and Holm-Šidák post hoc test. (**B**) Representative H&E staining of spinal cord sections harvested from chimeric WT and *Ctrp4^–/–^* mice showed inflammatory cell infiltration and demyelination at day 18 after immunization. Scale bars: 200 μm. (**C**) Flow-cytometric analysis of absolute cell numbers of CNS-infiltrating T cells (CD45^+^CD3^+^CD4^+^ T and CD45^+^CD3^+^CD8^+^ T) and B cells (CD45^+^CD3^–^CD19^+^) at 18 days after immunization. (**D**) Flow-cytometric analysis of absolute numbers of different CNS-infiltrating myeloid cells including monocytes (CD45^+^CD11b^+^Ly6C^+^), neutrophils (CD45^+^CD11b^+^Ly6G^+^), and DCs (CD45^+^CD11c^+^MHCII^+^) at 18 days after immunization. (**E**) Flow-cytometric analysis of absolute numbers of Th1 cells (IFN-γ^+^), Th17 cells (IL-17A^+^), and Tregs (CD25^+^Foxp3^+^) of CD4^+^ T cells infiltrated to the CNS harvested at 18 days after immunization. (**F**) Mean clinical scores of CTRP4*^fl/fl^* and CD4-cre CTRP4*^fl/fl^* mice were monitored after MOG immunization. Data are representative of 3 independent experiments. Statistical significance was determined using 2-way repeated-measures ANOVA. (**G**) H&E staining (left) and LFB staining (right) of spinal cord sections harvested from CTRP4*^fl/fl^* and CD4-cre CTRP4*^fl/fl^* mice at day 18 after EAE induction. Data are represented as mean ± SEM and are from 1 of 3 independent experiments with similar results. One-way ANOVA with Tukey’s post test (**C**–**E**). *P < 0.05; **P < 0.01; ***P < 0.0001.

Next, we determined whether CTRP4 executed its protective function primarily through immune cells by generating bone marrow chimeric mice. Notably, the irradiated WT mice reconstituted with WT bone marrow cells were protected from EAE and exhibited delayed disease onset compared with WT recipients transplanted with *Ctrp4^–/–^* bone marrow. Irradiated *Ctrp4^–/–^* recipient mice transplanted with WT bone marrow were more resistant to EAE induction than those transplanted with *Ctrp4^–/–^* bone marrow (Figure 3A). Histopathologic examination of affected spinal cord was further used to validate disease severity (Figure 3B). Likewise, *Ctrp4^–/–^* mice transplanted with *Ctrp4^–/–^*bone marrow had a significant increase in CD4^+^ T cell and Th17 cell infiltration in the CNS compared with *Ctrp4^–/–^* mice transplanted with WT bone marrow, suggesting that CTRP4 regulated inflammation primarily by controlling the recruitment of Th17 cells (Figure 3, C–E). Collectively, these results supported the essential roles of CTRP4 in immune cells rather than nonhematopoietic compartments.

To investigate the T cell–intrinsic effect of CTRP4, we generated T cell conditional CTRP4-KO mice (Supplemental Figure 3, A and B). When compared with littermate controls, CTRP4*^fl/fl^*CD4-cre mice developed significantly more severe EAE progression (Figure 3F). Increased immune cell infiltration and demyelination in spinal cord sections of CTRP4*^fl/fl^*CD4-cre mice were observed (Figure 3G), confirming the T cell–intrinsic role of CTRP4 in driving EAE-associated pathogenesis. Therefore, it might be interpreted that CTRP4 exerted neuroprotective effects through a T cell–intrinsic mechanism.

### CTRP4 suppresses IL-6–driven Th17 cell differentiation.

**Figure 4:**
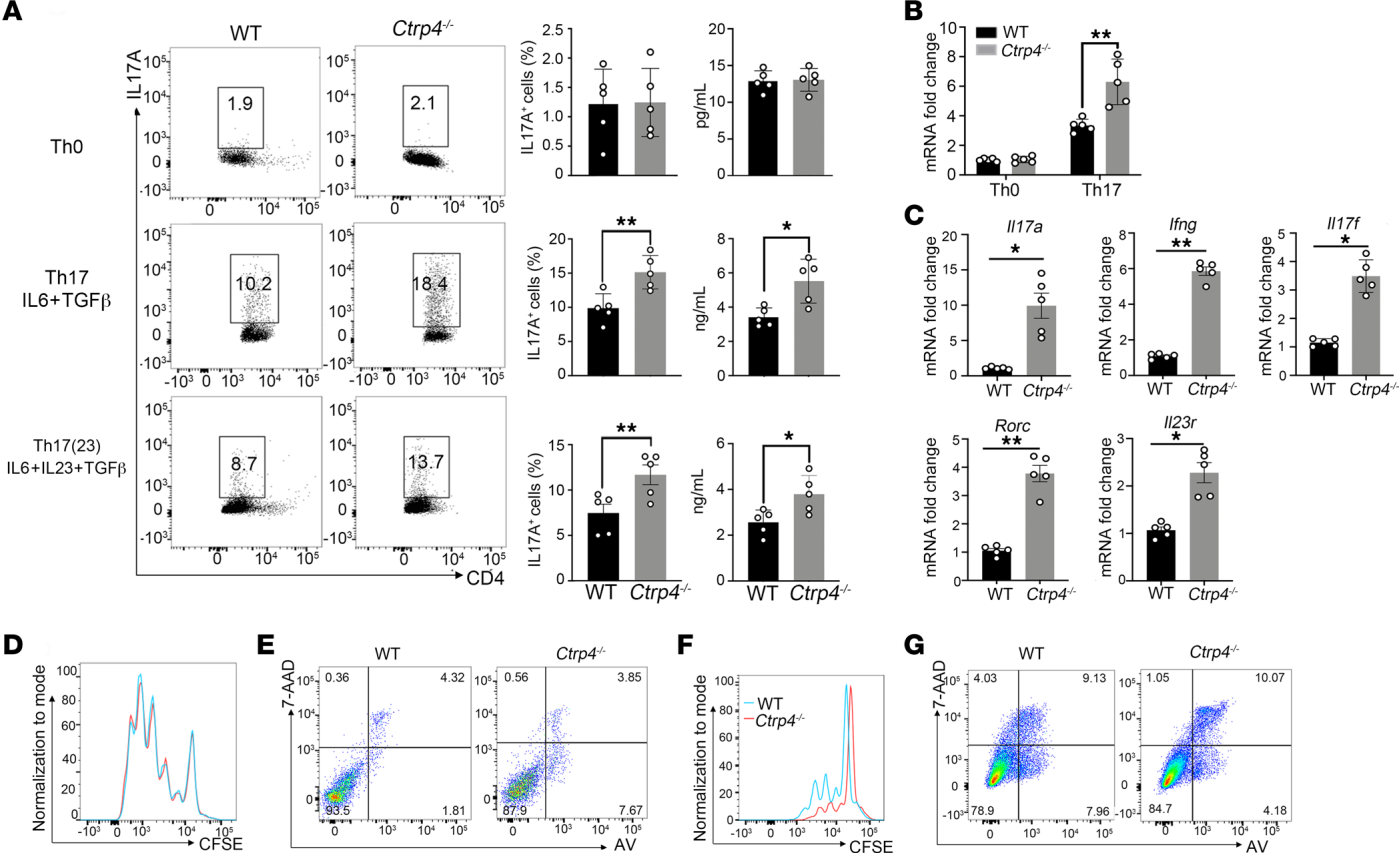
Naive *Ctrp4^–/–^* CD4^+^ T cells display an enhanced Th17 phenotype in vitro. (**A**) Naive CD4^+^CD62L^hi^CD44^lo^CD25^–^ T cells were sorted from WT and *Ctrp4^–/–^* mice and differentiated with no cytokine, TGF-β1 and IL-6, or IL-1β, IL-6, and IL-23. Numbers adjacent to outlined areas indicated the percentages of CD4^+^IL-17A^+^ cells. The production of IL-17A in the supernatants of different conditions was measured by ELISA (right). (**B** and **C**) Quantitative real-time PCR was used to quantify *Rorc* transcript expression in WT and *Ctrp4^–/–^* CD4^+^ T cells under Th0 or Th17 differentiation conditions (**B**) and quantify pathogenic Th17-associated gene expression of *Il17a*, *Il17f*, *Rorc*, *Ifng*, or *Il23r* mRNA in CD4^+^ T cells from *Ctrp4^–/–^* or WT mice (**C**). Values were normalized against *Gapdh*. (**D** and **E**) Representative flow plots of CD4^+^ T cells stimulated for 72 hours in the presence of TCR stimulations (anti-CD3/anti-CD28 antibodies). The proliferation of WT and *Ctrp4^–/–^* CD4^+^ T cells was measured by CFSE dilution assay (**D**). The apoptosis of WT and *Ctrp4^–/–^* CD4^+^ T cells was assessed by AV and 7-AAD staining (**E**). (**F** and **G**) Representative flow plots of naive CD4^+^ T cells after 72 hours in vitro Th17 cell–differentiated conditions to detect proliferation by CFSE dilution assay (**F**) or apoptosis by AV and 7-AAD staining (**G**). Data are represented as mean ± SEM and are from 1 of 3 independent experiments with similar results. (**A**–**C**) Statistical significance was determined using 2-tailed, unpaired Student’s *t* test or Mann-Whitney *U* test as appropriate after assessing for distribution. *P < 0.05; **P < 0.001.

In order to provide further insight into the mechanisms, we next investigated whether CTRP4 influenced the ability of naive CD4^+^ T cells toward Th17 differentiation or the ability of Th17 cells to expand, survive, or infiltrate into CNS, actions that are required for EAE onset and progression. Naive CD4^+^ T cells were cultured and analyzed under different Th17 cell–polarizing conditions. In nonpathogenic conditions, loss of *Ctrp4* greatly increased the number of IL-17A–producing cells and consequently the production of IL-17A (Figure 4A). The mRNA expression of transcription factors also supported the findings (Figure 4B). Moreover, we assessed whether CTRP4 deficiency affected the differentiation of pathogenic Th17 cells. When cells were cultured with IL-1β+IL-6+IL-23, a condition required for the acquisition of the pathogenic Th17 cell phenotype, *Ctrp4^–/–^* mice possessed a higher frequency of Th17 cells, leading to much higher levels of IL-17A production (Figure 4A). Consistent with the phenotypic data, *Ctrp4^–/–^* CD4^+^ T cells significantly upregulated Th17-associated gene signatures, including those of *Rorc*, *Il17a*, and *Il17f*, indicating that elevated IL-17 secretion was partially attributed to the altered transcriptional regulation. Of note, *Ctrp4* also affected the pathogenic capacity of Th17, as evidenced by the increased mRNA expression of *Il23r* and *Ifng* in *Ctrp4^–/–^* CD4^+^ T cells, which transcribed into cytokines essential for Th17 cell stability and pathogenicity (Figure 4C).

Next, we evaluated whether *Ctrp4* deficiency had an impact on T cell proliferative capacity. By flow cytometry analysis of CFSE dilution, we found *Ctrp4^–/–^* CD4^+^ T cells showed a similar proliferative capacity when stimulated with TCR activation or in the presence of Th17 cell–polarizing cytokines (Figure 4, D and E). Additionally, CTRP4-deficient CD4^+^ T cells had no effect on apoptotic induction (Figure 4, F and G). Encephalitogenic T cells express high levels of chemokine receptors to mediate the initial rolling and adhesion steps of transmigration and facilitate their recruitment to the CNS (16). Of note, the absence of *Ctrp4* in CD4^+^ T cells exhibited no alteration regarding CCR6, CCR2, CD49d, or CD29 expression, suggesting that *Ctrp4* did not alter the ability of Th17 cells to migrate to inflammation sites (Supplemental Figure 4, A and B). Consistent with this finding, the spinal cords of *Ctrp4*-deficient mice expressed similar mRNA levels of various chemokines mediating immune-cell recruitment compared with the levels of the WT cohort (Supplemental Figure 4C). Additionally, the absence of *Ctrp4* in Th17 cells had no effect on the expression of the key activation markers CD25, CD44, CD69, or CD103 (Supplemental Figure 4D). Therefore, it can be concluded that *Ctrp4* directed CD4^+^ T cell–fate choice toward differentiation into Th17 cells, ultimately leading to severe disease without affecting Th17 cell proliferation, apoptosis, migration, or activation of Th17 cells.

### CTRP4 acts as a negative regulator of IL-6R signaling.

**Figure 5:**
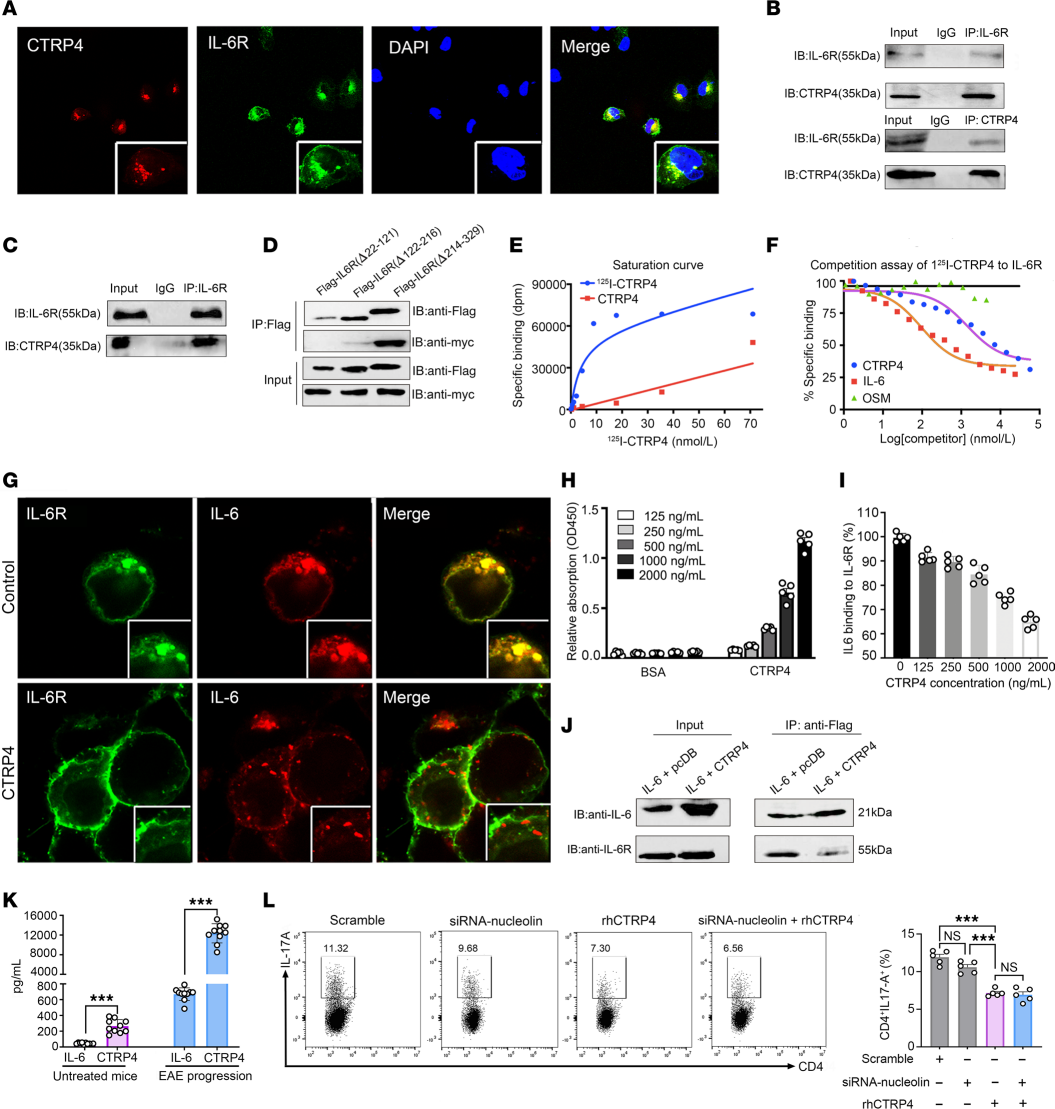
CTRP4 negatively regulates IL-6–induced STAT3 activation through the IL-6/IL-6R axis. (**A**) Jurkat cells transduced with lentivirus encoding pmCherry-CTRP4 and EGFP–IL-6R were observed using laser confocal microscopy. Higher magnification images of boxed areas of lower power images are provided. Original magnification, ×80; ×100. (**B**) Coimmunoprecipitation of membrane protein from Jurkat cell lysates by anti–IL-6R antibody and anti-CTRP4 antibody. (**C**) Cell membrane extracts from differentiated Th17 cells were coimmunoprecipitated by anti–IL-6R and then immunoblotted with anti-CTRP4 antibody (**D**) HEK293T were transfected with plasmid encoding Myc-tagged CTRP4 and Flag-tagged IL-6R truncated forms, followed by immunoblotting with indicated antibodies. (**E**) Equivalent quantities of Jurkat cell extract were incubated with serial dilutions of ^125^I-CTRP4 to calculate saturation curves. (**F**) Competitive binding assays were performed by addition of unlabeled rhCTRP4 to disturb the interaction between^125^I-CTRP4 and IL-6R. (**G**) HEK293T cells were transfected with plasmid encoding EGFP–IL-6R and pmCherry–IL-6 with or without rhCTRP4 to detect IL-6 binding to IL-6R. Original magnification, ×100. (**H**) rhCTRP4 or BSA was incubated with solid-phase 200 ng/mL IL-6R to detect the interaction between CTRP4 and IL-6R by ELISA. (**I**) Competitive blockade assays were performed adding 200 ng/mL IL-6 to compete with CTRP4 for binding to IL-6. (**J**) Jurkat cells transduced with retrovirus encoding Flag-tagged IL-6 or Flag-tagged IL-6 plus Myc-tagged CTRP4 were coimmunoprecipitated with anti-Flag antibody. (**K**) Serum levels of CTRP4 and IL-6 were determined by ELISA before model induction or at the peak of EAE. (**L**) Differentiation of CD4^+^ cells transfected with siRNA-nucleolin into Th17 cells was assessed in the presence or absence of rhCTRP4. (**H**–**L**) Data are represented as mean ± SEM obtained from independent experiments with similar results. The samples derived from the same experiment and gels/blots were processed in parallel. Statistical significance was determined using 2-tailed, unpaired Student’s *t* test or 1-way ANOVA with Tukey’s post test. ***P < 0.001.

Next, we assessed the involvement of IL-6 signaling in mediating the function of CTRP4 on Th17 cell differentiation. To verify this, Jurkat cells were cotransfected with pmCherry-CTRP4 and EGFP–IL-6R plasmids. The colocalized pattern was observed both in the cytoplasm and on the membrane (Figure 5A). Furthermore, the direct interaction was confirmed by coimmunoprecipitating in Jurkat cells (Figure 5B). Membrane-extracted protein from in vitro–differentiated Th17 cells was coimmunoprecipitated with anti-IL6R to detect the interactions between CTRP4 and IL-6R under physiological conditions. It was similar to what was seen in Jurkat (Figure 5C). To identify the region on IL-6R required for CTRP4 binding, HEK293T cells were cotransfected with Myc-tagged CTRP4 and different FLAG-tagged IL-6R truncated domains. Of note, IL-6R interaction with CTRP4 was dependent on the D3 domain (aa 214–329) of IL-6R (Figure 5D), which covers most of the IL-6 interface area (17).

To better evaluate interactive binding, the equivalent quantities of Jurkat cell membrane extract were incubated with serial dilution of ^125^I-CTRP4. Saturation-binding assays demonstrated that the direct binding affinity between CTRP4 and IL-6R was a K_D_ of 3.941 nM (Figure 5E). Moreover, the abilities of unlabeled CTRP4, IL-6, and OSM (another cytokine of the gp130 family) to replace ^125^I-labeled CTRP4 in the competition-binding assays were evaluated. The result demonstrated that unlabeled CTRP4 and IL-6 strongly competed with radio-iodinated CTRP4 to bind with IL-6R with IC_50_ values of 77.25 nM and 5.233 nM, respectively, while OSM did not exert a competitive effect (Figure 5F).

To investigate whether CTRP4 disturbed the formation of the IL-6/IL-6R complex, HEK293T cells were transfected with pmCherry-tagged IL-6 and EGFP-tagged IL-6R. Despite pronounced colocalization between IL-6 and IL-6R in cytoplasm and membrane, the pattern of colocalization was degenerated in the presence of CTRP4 (Figure 5G). Likewise, the results were confirmed by ELISA assays. In the plates precoated with IL-6R protein, CTRP4 bound to IL-6R in a dose-dependent manner (Figure 5H). As expected, the excess of IL-6 inhibited the binding of CTRP4 and IL-6R (Figure 5I). Consistent with this, coimmunoprecipitation further supported that CTRP4 interfered with the binding between IL-6 and IL-6R (Figure 5J). Although the binding affinity between IL-6R and CTRP4 was relatively low compared with the affinity of IL-6 for IL-6R, the circulating CTRP4 levels were higher by more than an order of magnitude than those of IL-6, which afforded more opportunity for CTRP4 to bind with IL-6 under disease states (Figure 5K). Given that nucleolin has been identified as the only known receptor for CTRP4, we assessed whether CTRP4 binding with nucleolin affected Th17 differentiation. After nucleolin expression was reduced with RNA interference, CTRP4 failed to alter the percentages of Th17 cells, excluding the possibility that nucleolin is involved in modulating Th17 differentiation (Figure 5L).

IL-6 also suppresses Treg generation by reducing TGF-β–induced Foxp3 expression (18). When cultured under induced Treg–polarizing (iTreg-polarizing) conditions, *Ctrp4^–/–^* CD4^+^ T cells were polarized into Tregs to the same extent as WT naive CD4^+^ T cells (Supplemental Figure 5A). Moreover, *Ctrp4^–/–^* Tregs suppressed effector CD4^+^ T cell proliferation in vitro with efficiency similar to that of WT Tregs (Supplemental Figure 5B). This is consistent with the observation that *Ctrp4^–/–^* EAE mice had unchanged percentages of CD4^+^CD25^+^Foxp3^+^ cells in CNS (Figure 2F). Thus, our data demonstrate that CTRP4 is dispensable for the generation and suppressive capacity of Tregs in vitro.

### Binding of CTRP4 to IL-6R suppresses IL-6–mediated STAT3 phosphorylation.

**Figure 6:**
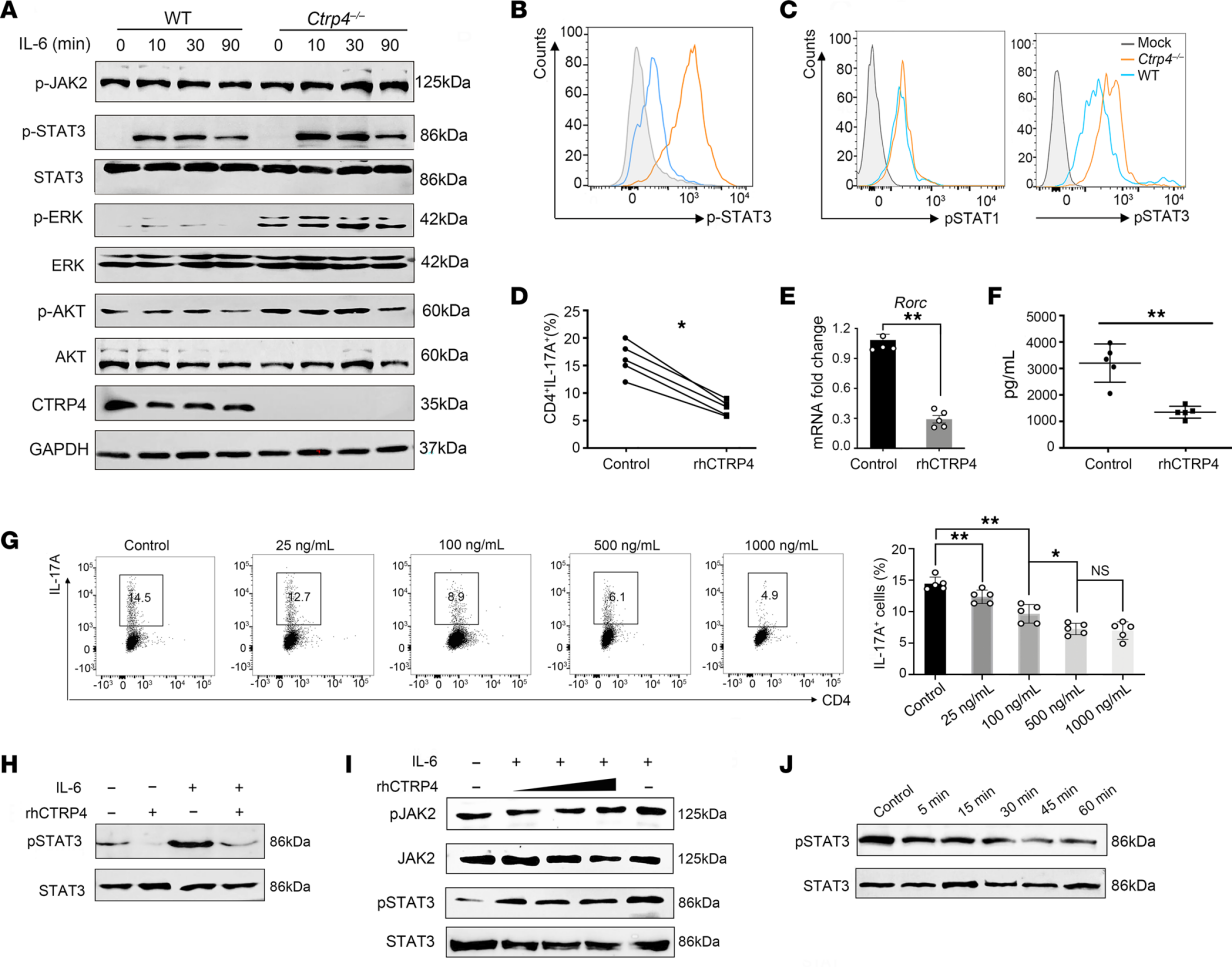
Reconstitution of CTRP4 inhibits IL-6–mediated STAT3 activation. (**A**) Purified CD4^+^ T cells from *Ctrp4^–/–^* and WT mice were stimulated with IL-6 (100 ng/mL) for indicated times. Lysates were subjected to Western blot analysis for indicated antibodies. (**B**) Freshly isolated WT and *Ctrp4^–/–^* CD4^+^ T cells were treated with IL-6 (100 ng/mL) for 30 minutes. Levels of p-STAT3 were determined by flow cytometry. (**C**) Representative flow cytometry analysis of p-STAT3 and p-STAT1 of CD4^+^ T cells from *Ctrp4^–/–^* and WT EAE-induced mice at the peak of disease. (**D**–**F**) WT CD4^+^ T cells were polarized to Th17. The intracellular IL-17A was analyzed by flow cytometry (**D**), and gene expression levels of *Rorc* mRNA were analyzed by quantitative real-time PCR (**E**). Supernatants were collected to determine levels of IL-17A by ELISA (**F**). (**G**) Purified WT naive CD4^+^ T cells were polarized under Th17 conditions with indicated doses of rhCTRP4. Quantification of the percentages of CD4^+^IL17A^+^ cells was analyzed by flow cytometry. (**H**) Purified WT CD4^+^ cells were activated with IL-6 (10 ng/mL) or rhCTRP4 (100 ng/mL) alone or treated with IL-6 prior to treatment with rhCTRP4 for 1 hour or without treatment as control. Western blot was performed to analyze the activation of STAT3. (**I**) Purified WT CD4^+^ cells were activated with IL-6 or with IL-6 pretreated with various concentrations of rhCTRP4 (100 ng/mL, 500ng/mL, and 1,000 ng/mL) followed by Western blot to analyze the activation of STAT3 and JAK2. The samples derived from the same experiment, and gels/blots were processed in parallel. Representative of 3 independent experiments with 5 mice per experiment. (**J**) Purified WT CD4^+^ cells were activated with IL-6 (10 ng/mL) prior to treatment with rhCTRP4 (100 ng/mL) for the indicated times. Time-dependent changes in the levels of p-STAT3 were evaluated by Western blot. Data are represented as mean ± SEM and are from 1 of 3 independent experiments with similar results. (**D**–**F**) Statistical significance was determined using paired Student’s *t* test; (**G**) statistical significance was determined using 1-way ANOVA with Tukey’s post test. *P < 0.05; **P < 0.01.

IL-6 promotes Th17 cell differentiation through the activation of STAT3, especially at tyrosine 705 (19), which prompted us to evaluate the inhibitory effect of CTRP4 on JAK/STAT3 signaling. First, we found that the expression of IL-6R was comparable between WT and *Ctrp4*-deficient CD4^+^ T cells upon TCR stimulation (Supplemental Figure 6, A and B). Notably, increased expression levels of phosphorylated STAT3 (p-STAT3) in *Ctrp4^–/–^* CD4^+^ T cells stimulated with physiological concentrations of IL-6 were observed, and the activation effect was observed in a more pronounced fashion after stimulation with IL-6 (Figure 6A). This was supported by enhanced STAT3 phosphorylation in *Ctrp4^–/–^* CD4^+^ T cells when exposed to IL-6 compared with that in WT CD4^+^ T cells by flow cytometry (Figure 6B). In terms of other associated signaling molecules, p-JAK2, p-ERK, and p-Akt also showed higher expression in the *Ctrp4^–/–^* CD4^+^ T cells in response to IL-6, suggesting that the IL-6/STAT3 signaling pathway was constitutively hyperactivated after the loss of CTRP4 in CD4^+^ T cells (Figure 6A). To address the molecular mechanisms by which CTRP4 inhibited the progression of EAE in vivo, we first measured the expression of IL-6R and gp130 in an EAE model. Flow cytometry analysis showed that the expression levels of IL-6R and gp130 in the CD4^+^ T cells isolated either from inflammation sites or peripheral lymphoid organs remained comparable between groups (Supplemental Figure 6C). Notably, the deficiency of CTRP4 in CD4^+^ T cells resulted in an increase in Y705-phosphorylated STAT3 in the spinal cord tissue of EAE mice compared with the levels of CD4^+^ T cells in WT mice, while the abundance of p-STAT1 was equivalent (Figure 6C). In addition, the *Ctrp4^–/–^* CD4^+^ cells were treated with IL-27 for indicated periods to verify whether CTRP4 only responded to IL-6 rather than other gp130 family cytokines. The results showed that the JAK/STAT3 signaling pathway was activated in *Ctrp4^–/–^* CD4^+^ T cells, similarly to what was observed in WT cells (Supplemental Figure 7A).

We further verified whether the addition of rhCTRP4 protein impaired Th17 cell differentiation. A decrease in the percentages of Th17 was observed in vitro with rhCTRP4 treatment (Figure 6D). Under Th17 cell–differentiating conditions, CD4^+^ T cells treated with rhCTRP4 showed significantly reduced *Rorc* transcript levels (Figure 6E) and IL-17A production in the supernatant (Figure 6F). Furthermore, naive CD4^+^ T cells with various concentrations of rhCTRP4 under Th17-polarizing condition were evaluated and it was found that higher doses of rhCTRP4 inhibited Th17 cell differentiation to a larger extent, suggesting that rhCTRP4 impaired Th17 differentiation in a dose-dependent manner (Figure 6G). Consistent with the aforementioned results, the pretreatment of naive CD4^+^ T cells with rhCTRP4 abrogated STAT3 activation in response to IL-6 (Figure 6H). Accordingly, rhCTRP4 also attenuated IL-6–induced phosphorylation of JAK2 and STAT3 to normal levels in a dose-dependent manner (Figure 6I). IL-6–triggered STAT3 phosphorylation activation was abolished as early as 5 minutes after exposure to rhCTRP4, and the inhibitory effect was sustained for at least 60 minutes (Figure 6J). Taken together, these results further confirm that CTRP4 negatively regulates IL6/STAT3 signaling.

**Figure 7:**
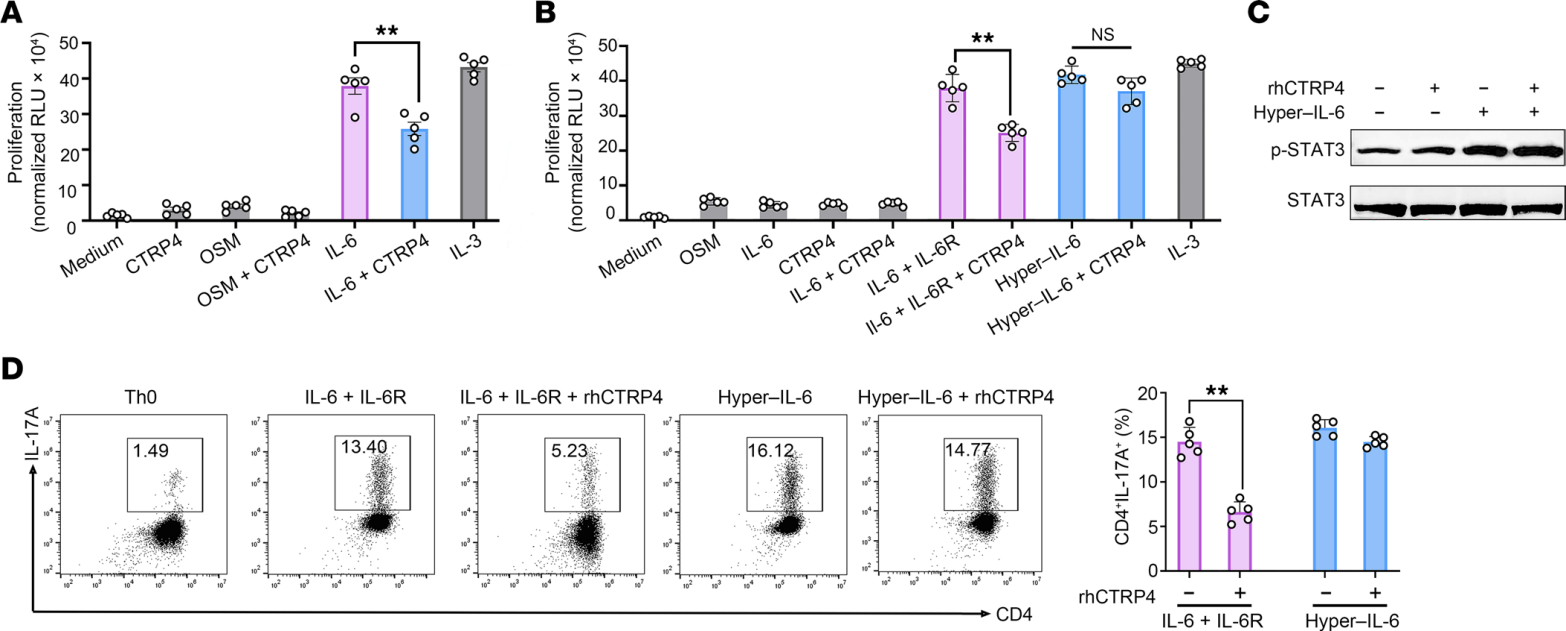
Mechanism underlying the inhibition of Th17 cell differentiation by CTRP4. (**A** and **B**) Proliferative response of Ba/F3–gp130–IL-6R cells cultured for 2 days in the presence of exogenous rhCTRP4 (100 ng/mL), OSM (100 ng/mL), IL-6 (10 ng/mL), IL-3 (10 ng/mL), rhCTRP4 plus IL-6, or OSM plus rhCTRP4, or left untreated (**A**). (**B**) Proliferative response of Ba/F3-gp130 cells cultured for 2 days with OSM (100 ng/mL), IL-6 (10 ng/mL), rhCTRP4 (100 ng/mL), hyper–IL-6 (10 ng/mL), a combination of IL-6 (10 ng/mL) and IL-6R (10 ng/mL), a combination of IL-6 and IL-6R plus rhCTRP4, hyper–IL-6 plus rhCTRP4, or IL-3 (10 ng/mL). The proliferation in indicated culture conditions was determined by the colorimetric CCK8 assay. Results are shown as RLUs and normalized to the growth of cells cultured in medium. (**C**) Purified WT CD4^+^ cells were activated with hyper–IL-6 (10 ng/mL) prior to treatment with rhCTRP4 for 1 hour or without treatment as control. Western blot was performed to analyze the activation of STAT3. (**D**) Naive CD4^+^ T cells were differentiated toward Th17 cells with TGF-β plus the combination of IL-6 (10 ng/mL) and IL-6R (10 ng/mL) in the presence or absence of rhCTRP4 (100 ng/mL) or differentiated toward Th17 cells with TGF-β and hyper–IL-6 (10 ng/mL) in the presence or absence of rhCTRP4 (100 ng/mL). Data are represented as mean ± SEM and are from 1 of 3 independent experiments with similar results. (**A**, **B**, and **D**) Statistical significance was determined using 1-way ANOVA with Tukey’s post test.**P < 0.01.

Previous studies have shown that the IL-6 signaling cascade is initiated by the binding of IL-6 to membrane-bound IL-6R and gp130, which is called classical signaling (20). Then we used Ba/F3 cell, an IL-3–dependent mouse pro–B cell line lacking both endogenous IL-6R and gp130 expression (21), to establish the cell lines with stable expression of both gp130 and IL-6R (Ba/F3–gp130–IL-6R) or expression of gp130 only (Ba/F3/gp130) through the lentiviral system (Supplemental Figure 7, B and C) and then analyzed their proliferation response to IL-6 signaling. Proliferation of Ba/F3–gp130–IL-6R relies on IL-6–mediated classical signaling, while proliferation of Ba/F3-gp130 relies on both IL-6 and IL-6R–mediated trans-signaling. First of all, IL-6 alone significantly promoted the proliferation of Ba/F3-gp130–IL-6R cells, whereas the IL-6–mediated proliferation rate was reduced in the presence of rhCTRP4, suggesting that CTRP4 affected classical IL-6 signaling (Figure 7A). Afterwards, we investigated the roles of rhCTRP4 on IL-6 trans-signaling. Ba/F3-gp130 was not responsive to IL-6 and sufficiently restored growth in response to a combination of IL-6 plus sIL-6R. Exogenous CTRP4 significantly suppressed the proliferation of Ba/F3-gp130 cells induced by the combination of IL-6 and IL-6R (Figure 7B), indicating that CTRP4 retains the ability to respond to IL-6 trans-signaling.

Next, we aimed to get deep insight of the inhibitory effect of CTRP4 on the already formed IL-6/IL-6R complex. Experimentally, Ba/F3-gp130 cells were pretreated with hyper–IL-6, which mimics the preassembled IL-6/IL-6R complex, in the presence or absence of rhCTRP4. The strong proliferative response induced by hyper–IL-6 was not impaired after the addition of CTRP4 (Figure 7B). Western blot analysis showed a remarkable increase of p-STAT3 in CD4^+^ T cells after treatment with hyper–IL-6, whereas the addition of CTRP4 was not able to inhibit the activation of STAT3 induced by hyper–IL-6 (Figure 7C). To ascertain whether CTRP4 was functionally important for the already formed IL-6/IL-6R complex, naive CD4^+^ cells were differentiated under the Th17 cell–polarizing condition with hyper–IL-6 or with the combination of IL-6 and IL-6R. The results showed that the frequency of Th17 cells was moderately increased after hyper–IL-6 treatment and the presence of rhCTRP4 inhibited the generation of Th17 when treated with the mixture of IL-6 and IL-6R protein. However, the presence of CTRP4 showed no significant effects on inhibiting Th17 differentiation when treated with the mixture containing the IL-6/IL-6Rα complex (Figure 7D). Hence, these results indicate that CTRP4 was unable to disrupt the already formed IL-6/IL-6Rα complex.

### rhCTRP4 treatment reduces neuroinflammation in EAE.

**Figure 8:**
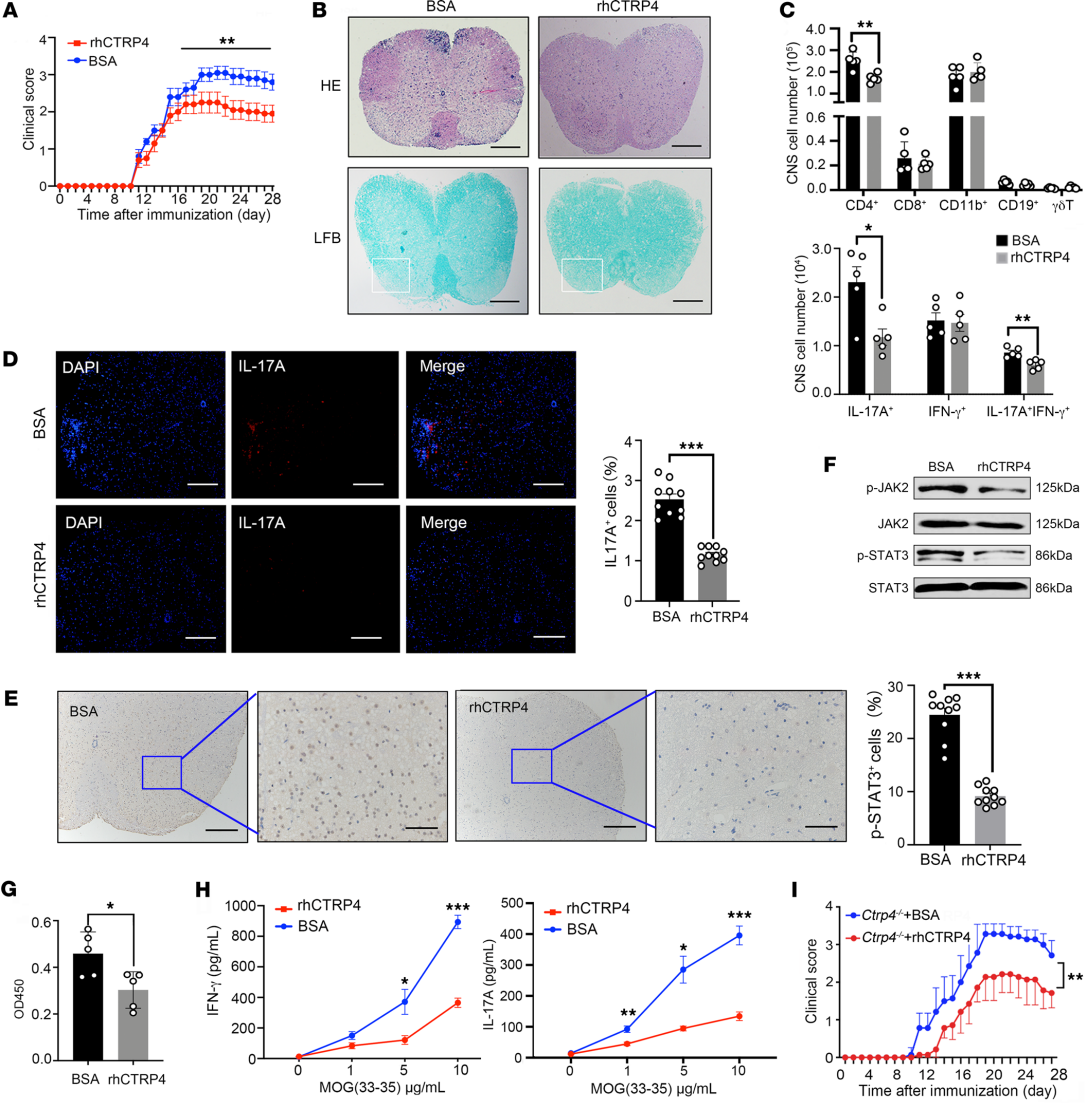
In vivo administration of rhCTRP4 attenuates clinical severity. (**A**) After EAE induction of WT mice, 500 ng/mice rhCTRP4 or control (BSA) were administered intraperitoneally every day starting at disease onset on day 9 after immunization until sacrificed. The clinical scores were monitored daily and are depicted (*n* = 10/group). (**B**) Representative images of H&E and LFB staining on spinal cord sections of mice at the peak of EAE. Scale bars: 200 μm. (**C**) CNS monocytes were harvested on day 18. The absolute cell numbers of indicated CNS-infiltrating cell populations (gated on CD45^+^) or the absolute numbers of CD4^+^IFN-γ^+^, CD4^+^IL-17A^+^, and CD4^+^IL-17A^+^IFN-γ^+^ in CNS were analyzed. (**D**) Representative immunofluorescent images and quantification of IL-17A^+^ cells in the spinal cord of indicated mice at day 18 after immunization. Nuclei were counterstained with DAPI. Scale bars: 100 μm. (**E**) Representative immunohistochemistry detection and quantification of p-STAT3(Y705) in the spinal cords of indicated mice at day 18 after immunization. Scale bars: 100 μm. (**F**) CD4^+^ T cells were isolated from *Ctrp4^–/–^* and WT mice 18 days after EAE induction and the activity phosphorylation of STAT3 and JAK2 was detected by Western blot. (**G** and **H**) dLN CD4^+^ T cells isolated from rhCTRP4-treated group or control group were expanded with irradiated autologous-presenting cells plus 10 μg/mL (**G**) or indicated concentrations (**H**) of MOG_35–55_ peptide for 72 hours and subjected to cell-proliferation assay to determine T cell recall response based on BrdU assay (**G**) or quantitate the productions of IL-17A and IFN-γ (**H**). (**I**) *Ctrp4^–/–^* female mice were immunized with MOG_35–55_ peptide to induce EAE; 500 ng/mice rhCTRP4 or control (BSA) was administered intraperitoneally every day starting at disease onset on day 9 after immunization until sacrificed to restore the level of CTRP4. Clinical scores are depicted (*n* = 8/group). Data are represented as mean ± SEM and are from 1 of 3 independent experiments with similar results. (**A** and **I**) Statistical significance was determined using 2-way repeated-measures ANOVA. Data were analyzed by unpaired Student’s *t* test or Mann-Whitney *U* test as appropriate after assessing for distribution (**C**–**E**, **G**, and **H**). *P < 0.05; **P < 0.01; ***P < 0.001.

Next, we wondered whether rhCTRP4 was effective in alleviating established EAE disease in WT mice by daily intraperitoneal injection of rhCTRP4 starting from day 9 after immunization, a time point widely considered to represent the onset of symptoms. In comparison with BSA treatment, rhCTRP4 treatment demonstrated significantly increased therapeutic efficacy and reduced EAE severity (Figure 8A). The histological analysis revealed that the administration of rhCTRP4 was accompanied by decreased inflammation and demyelination in the affected spinal cord (Figure 8B). In the CNS, the number of CD4^+^ T cells, particularly CD4^+^IL-17A^+^ and CD4^+^IFN-γ^+^IL-17A^+^ cells, were dramatically reduced by therapeutic rhCTRP4 administration (Figure 8C). Similar results were observed by immunofluorescence staining of IL-17A (Figure 8D). The reduced nuclear translocation of p-STAT3 and STAT3 phosphorylation in EAE mice treated with rhCTRP4 also supported the CTRP4-mediated inhibitory effects on STAT3 activation in vivo (Figure 8, E and F). When T cells from primed mice were challenged with the MOG peptide to detect reactivity toward the antigen, the T cells from EAE mice treated with rhCTRP4 displayed a markedly dampened proliferative response (Figure 8G) along with reduced IL-17A and IFN-γ generation (Figure 8H).

Given that CTRP4-deficient mice showed more aggressive pathogenesis, we reasoned whether the addition of rhCTRP4 rescued the severe disease phenotype. After intraperitoneal administration with rhCTRP4, CTRP4-deficient mice developed milder EAE, as demonstrated by lower clinical scores (Figure 8I). In summary, therapeutic delivery of rhCTRP4 ameliorated the clinical severity of EAE associated with reduced encephalitogenic effector T cell responses.

### CTRP4 directly inhibits IL-6 signaling in vivo.

**Figure 9:**
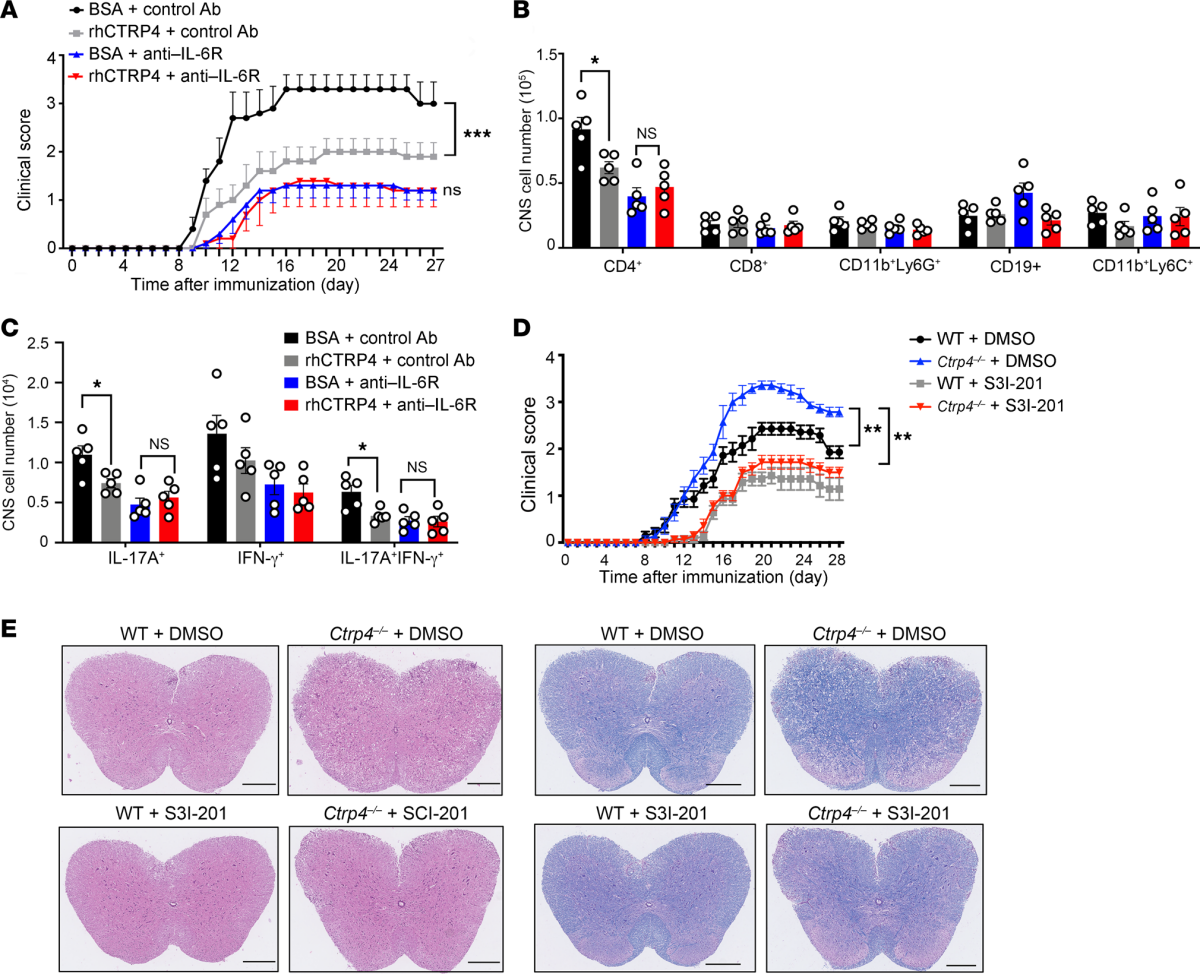
IL-6 signaling blockade abrogates the protective effects of CTRP4 in EAE. (**A**) WT mice were intraperitoneally injected with anti–IL-6R or control IgG on immunization days –1, 3, 7, 11, and 15(*n* = 5 mice/group). After EAE induction, mice were treated with rhCTRP4 or BSA daily from day 0 to day 27. Mean clinical scores show progression of disease. (**B**) CNS monocytes were harvested on day 18 and quantified as absolute cell numbers of indicated CNS-infiltrating cell populations gated on CD45^+^. (**C**) CNS monocytes were harvested on day 18 and quantified as absolute numbers of CD4^+^IFN-γ^+^, CD4^+^IL-17A^+^, and CD4^+^IL-17A^+^IFN-γ^+^ in CNS after stimulating with PMA and inomycin with GolgiPlug for 5 hours. (**D**) *Ctrp4^–/–^* and WT mice subjected to MOG-induced EAE were treated with selective STAT3 inhibitor S3I-201 (10 mg/kg/d dissolved in 20%DMSO/80% corn oil). Control mice were injected with equal volumes of vehicle. Each group was monitored and scored daily (*n* = 7/group). (**E**) Representative images of H&E staining and LFB staining of spinal cord sections show inflammatory cell infiltration and demyelination, respectively. Scale bar: 200 μm. Data are represented as mean ± SEM and were analyzed by 1-way ANOVA with Tukey’s post test (**B** and **C**). Two-way repeated measures ANOVA and Holm-Šidák post hoc test (**A** and **D**).*P < 0.05; **P < 0.01.

To investigate whether CTRP4 directly inhibited IL-6 function in vivo, EAE mice were injected intraperitoneally with rhCTRP4 or vehicle as well as with the neutralizing anti–IL-6R antibody. We found mice treated with anti–IL-6R antibody developed significantly milder disease, demonstrated by delayed disease onset, relative to that of control mice injected with IgG, suggesting that IL-6R blockade contributed to the remission of EAE as expected. However, the protective effects of rhCTRP4 were abrogated in the absence of IL-6R (Figure 9A), as evidenced by the similar clinical severity of mice treated with anti–IL-6R antibody in the presence or absence of rhCTRP4. Furthermore, the reduction of CD4^+^ T cells caused by rhCTRP4 administration was abolished by the addition of anti–IL-6R (Figure 9B). For subsets of CNS-infiltrating CD4^+^ T cells, the decreased tendency of Th17 in CNS-infiltrating CD4^+^ T cells in mice treated with rhCTRP4 disappeared in the presence of anti–IL-6R (Figure 9C), confirming the importance of the interaction of CTRP4 with IL-6R in vivo.

Next, we investigated whether the suppressive effect of CTRP4 on STAT3 activation contributed to the protection of the host resistant to EAE. To this end, a STAT3 small-molecule inhibitor, S3I-201 (22), was used to suppress STAT3 signaling in vivo and we found S3I-201 administration improved the severe symptoms caused by CTRP4 deficiency (Figure 9D), as indicated by a reduction of immune cells infiltrated into the spinal cord and decreased demyelination (Figure 9E). Similarly to the inhibitory effects of CTRP4 on STAT3 activation in vitro, CTRP4 ameliorated disease by inhibiting STAT3 in vivo.

### Mice transferred with MOG-reactive T cells that expand in the presence of rhCTRP4 develop mild EAE.

**Figure 10:**
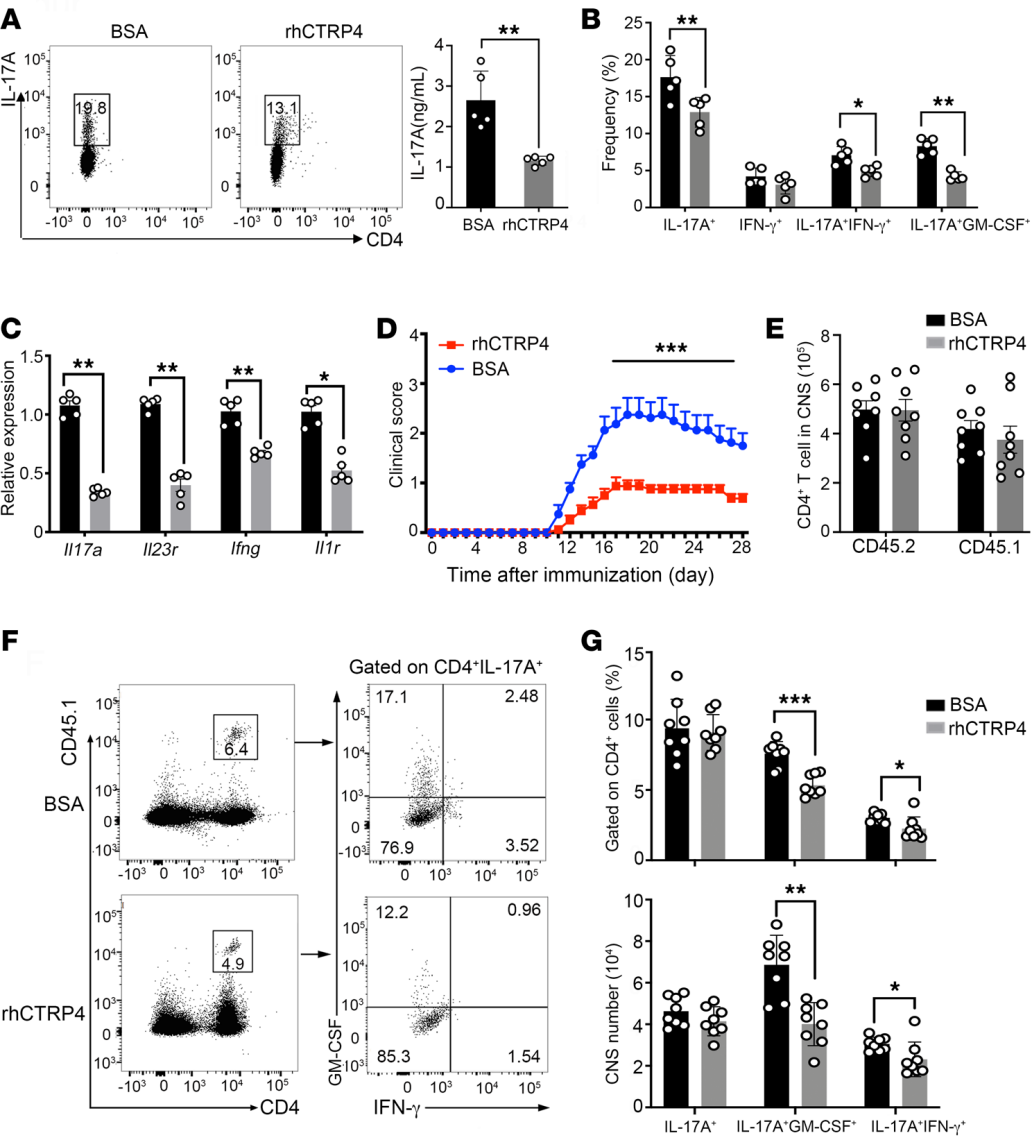
Mice transferred with MOG-reactive T cells that expand in the presence of rhCTRP4 develop mild EAE. (**A**) Lymphocytes from the dLNs of B6.SJL mice (CD45.1^+^) that were previously immunized with MOG_35–55_ in CFA were rechallenged with the MOG_35–55_ peptide in the presence of BSA or rhCTRP4 under Th17 polarization conditions. The intracellular IL-17A was analyzed via flow cytometry (left), and the production of IL-17A in the supernatants was measured by ELISA (right). (**B**) After PMA/ionomycin stimulation for 5 hours, the representative FACS plots and the frequency of CD4^+^IL-17A^+^, CD4^+^IFN-γ^+^, CD4^+^IL-17A^+^IFN-γ^+^, and CD4^+^IL-17A^+^GM-CSF^+^ before the time of adoptive transfer were determined. (**C**) Ex vivo–expanded MOG-specific CD4^+^ T cells under Th17 polarization conditions in the presence of BSA or rhCTRP4 were analyzed for the expression of indicated genes by quantitative PCR. The values were normalized against *gapdh*. (**D**–**G**) Ex vivo–expanded MOG-specific CD4^+^ pretreated with BSA or rhCTRP4 were transferred into irradiated congenic recipients (CD45.2) to induce EAE. (**D**) Clinical scores of EAE progression were monitored daily. (**E**) The mononuclear cells isolated from brain and spinal cord at disease peak stage were analyzed by flow cytometry. Absolute numbers of CD4^+^ T cells of donor and recipient mice in CNS were analyzed. Gated CD45.1^+^CD4^+^IL-17^+^ T cells were analyzed for the production of IFN-γ and GM-CSF. Representative contour plots (**F**) show the percentages and absolute numbers of CD4^+^IL-17^+^IFN-γ^+^, CD4^+^IL-17^+^GM-CSF^+^ donor cells in the CNS (**G**). Data are represented as mean ± SEM and are from 1 of 3 independent experiments with similar results. Two-way repeated-measures ANOVA was used for **D**. Statistical significance was determined using unpaired Student’s *t* test or Mann-Whitney *U* test for **A**–**C**, **E**, and **G**. *P < 0.05; **P < 0.01; ***P < 0.001.

To further assess whether CTRP4 impaired the encephalitogenic potential of effector T cells in vivo, we used an adoptive transfer model of EAE. The in vitro polarization of the CD4^+^ T cells treated with rhCTRP4 showed a significant reduction in the frequency of MOG-specific Th17 cells and IL-17A secretion in the culture supernatants compared with that stimulated with BSA (Figure 10A). With respect to the coexpression of inflammatory cytokine in Th17 cells, rhCTRP4 downregulated the production of GM-CSF and IFN-γ (Figure 10B), which were associated with the encephalitogenic potential of the Th17 cells to elicit neuroinflammation. Consistent with this, rhCTRP4 significantly decreased *Il17a*, *Ifng*, *Il1r*, and *Il23r* mRNA transcripts in MOG-reactive CD4^+^ T cells (Figure 10C).

The expanded MOG-reactive CD4^+^ T cells in the presence of rhCTRP4 were transferred into irradiated recipient mice, and the recipient mice exhibited significantly milder symptoms compared with mice receiving MOG-reactive CD4^+^ T cells stimulated with BSA (Figure 10D). We observed that the numbers of CD45.2^+^CD4^+^ host T cells and CD45.1^+^ CD4^+^ donor T cells were comparable in CNS between the 2 groups (Figure 10E). Moreover, the number of Th17 cells among the CD45.1^+^ donor T cell population from mice receiving donor T cells stimulated with rhCTRP4 were similar to those of the donor T cells stimulated with BSA (Figure 10G). However, we observed a dramatic difference in the pattern of pathogenic cytokines of Th17 cells from the recipient mice receiving T cells treated with exogenous CTRP4, coexpressing higher GM-CSF and IFN-γ (Figure 10, F and G). Collectively, we inferred that pretreatment with rhCTRP4 during the ex vivo expansion of MOG-reactive CD4^+^ T cells rendered the T cells less encephalitogenic in inducing autoimmune CNS inflammation.

## Discussion

Herein, we provided comprehensive evidence to confirm the immunomodulatory properties of CTRP4 in modulating T cell function during the pathophysiology of EAE: (a) the initial priming of T cells in peripheral lymphoid organs; (b) differentiation of primed T cells toward Th17 cells; and (c) migration of pathogenic T cells into the CNS leading to the onset of symptoms. In addition, we found the mechanism of reduction of IL-6 activity by CTRP4 was not through regulating IL-6R or gp130 expression, but through direct binding with IL-6R, leading to the suppression of IL-6–induced activation of STAT3, which is an essential regulator of the lineage commitment to Th17 cells (23). Of note, McGeachy et al. pointed toward a critical role for STAT3 in maintaining the capacity of Th17 cells to produce cytokine in response to antigenic stimuli compared with stimulation with PMA and ionomycin (24). This is consistent with our results showing that CD4^+^ T cells from *Ctrp4*-deficient mice enhanced cytokine production after restimulation with MOG peptides (Figure 2I) and showed no change in proliferative capacity when stimulated with TCR activation (Figure 4D). CTRP4 may also play a role in the disease by regulating other cell types, such as macrophages infiltrating in CNS (Figure 2D). Additional work is needed for a more comprehensive understanding of the universal role of CTRP4 in other IL-6R–expressing cells.

The biological effects of IL-6 are highly complex, as they are mediated via multiple pathways. Notably, Casey Weaver’s findings indicated that the role of IL-6 signaling is beyond the inductive phase of Th17 and is thought to be responsible for Th17 cell maintenance (25). Our results also show that CTRP4 added to ex vivo culture was able to suppress Th17 responses and that MOG-reactive CD4^+^ T cells expanded in the presence of rhCTRP4 induced milder symptoms after being adoptively transferred into irradiated recipients. Together, our data were consistent with the finding from the Weaver group’s report that IL-6R–deficient TH17 cells rapidly lost their Th17 phenotype. Based on these results, we inferred that CTRP4 disrupted the Th17 generation via both the classical and trans-signaling pathways. However, more detailed evidence is needed for proof.

Although Mufazalov et al. claimed that IL-6R is the only biologically relevant receptor for IL-6 in mice (26), it should be noted that Hua Yu et al. demonstrated IL-6 is also able to bind to CD5 in B1a independently of IL-6R (27). Of note, CD5 expression was restricted to B1a cells, implying that the IL-6–CD5 module was not common. In addition, IL-6–overexpressing mice developed a lethal immune dysregulation syndrome with massively infiltrated CD11b^+^ myeloid cells expressing robust IL-6Ra, but no CD5. This may partly explain why the interaction between CD5 and IL-6 failed to work. In addition, Wael El-Rifai et al. found that TFF1 interfered with IL-6R and further compromised the formation of the IL-6–mediated IL-6Rα/gp130 complex, which played a protective role in mucosal integrity against gastric tumorigenesis (28). Furthermore, other antagonists targeting gp130 led to the disturbance of the IL-6/IL-6R/gp130 system. For instance, IL-27p28 is a natural antagonist for blocking gp130-mediated signaling via interaction at a lower affinity compared with IL-6R/gp130 interaction. Furthermore, tumor necrosis factor receptor–associated factor 5 (TRAF5) has also been reported as constitutively binding to gp130, antagonizing IL-6–driven activation of STAT3 (29). Indeed, our work is also of importance for filling out current knowledge of the IL-6/IL-6R/gp130 system.

It is well established that the administration of the IL-6 receptor antagonist tocilizumab holds promise for treating potentially fatal cytokine release syndrome observed in CAR-T therapy (30) or SARS-CoV-2 infection (31), underscoring the importance of defining regulators of IL-6 homeostasis. Different IL-6 signaling modes could be distinguished by circulating forms of gp130. IL-6 forms a complex with the sIL-6R and gp130 in blood to prolong its half-life. Thus, sIL-6R and sgp130 are thought to create a biological buffer system to regulate the IL-6 biological effect by capturing free IL-6 and neutralizing rapidly (32). Our study found that CTRP4 could be a part of the broader biological buffer system by competitively binding IL-6R, even though it has a lower binding affinity. Further work is required to determine whether at least a portion of IL-6R’s biological functions are contributed to by its ability to interact with CTRP4. Besides, the IL-6–blockade strategy failed to show efficacy for all autoimmune diseases, suggesting that new insights into the understanding of the IL-6 system could help to promote the development of therapeutic drugs.

## Methods

### Experimental mice.

The generation of *Ctrp4^–/–^* mice has been previously described (23). The CTRP4*^fl/fl^* mice were from Li Yingxian’s lab. CD4-Cre and EIIa-Cre mouse strains came from the Jackson Laboratory. B6.SJL mice were purchased from the Jackson Laboratory. Six- to twelve-week-old mice were used for most of the experiments. Age- and sex-matched littermates with the appropriate genotypes were used as controls. All mice were bred and maintained under specific pathogen–free (SPF) conditions in an animal facility.

### EAE model establishment.

The MOG_35–55_ peptide with the aa sequence MEVGWYRSPFSRVVHLYRNGK was synthesized by Synpeptide. Mice were subcutaneously immunized with 200 μg of MOG_35–55_ emulsified with incomplete Freund’s adjuvant (catalog F5506, MilliporeSigma) containing 5 mg/mL heat-killed *Mycobacterium tuberculosis* (catalog 231141, BD Biosciences), followed by a tail-vein injection of 200 ng of pertussis toxin (catalog 179B, List Biological Laboratories) on day 0. Intraperitoneal injection of 200 ng of pertussis toxin was administered on day 2. To verify the effect of rhCTRP4 in EAE, WT or *Ctrp4^–/–^* mice were immunized and then administrated intraperitoneally 500 ng/mice rhCTRP4 or vehicle daily starting on day 9 after immunization until sacrificed. The mice were monitored daily for clinical signs of disease on a scale of 1–4 as follows (33):0, no clinical symptoms; 1, limp tail without hind-limb weakness; 1.5, tail paralysis and waddling gait; 2, partial hind-limb weakness; 2.5, paralysis of 1 hind limb; 3, completely paralyzed hind legs; 3.5, complete hind-limb and partial forelimb paralysis; and 4, complete paralysis accompanied by urinary or fecal incontinence. For pharmacological inhibition of STAT3, STAT3 inhibitor S3I-201 (Selleck, catalog S1155) was injected intraperitoneally at 5 mg/kg every 2 days. For IL-6R blockade, WT mice were intraperitoneally injected with 10 mg/kg of anti–IL-6R (clone 15A7; BioXCell) or IgG1 (clone LTF-2; BioXCell) on day 0 before EAE induction or days 7, 14, and 21 after immunization. rhCTRP4 or BSA was administrated intraperitoneally in mice pretreated with neutralization antibodies every other day, starting on day 1 after immunization.

### Induction of EAE by passive transfer of pathogenic CD4^+^ T cells.

B6.SJL donor mice (CD45.1^+^) were induced as an EAE model. Ten days later, dLNs were isolated and then restimulated with 50 μg/mL of MOG peptide and Th17 cell–polarizing factors (20 ng/mL rhIL-6, 20 ng/mL rhIL-23, 20 ng/ml IL-1β and 10 μg/mL anti-IL4, and 10 μg/mL anti–IFN-γ) to generate MOG-specific Th17 cells. After 4 days in culture, the resting cells were sorted with anti-CD4 microbeads (Miltenyi Biotec), the number of CD4^+^ T cells was calculated, and then 1 × 10^6^ CD4^+^ T cells per mouse were intravenously injected into irradiated C57BL/6J recipient mice (4 Gy). Next, the mice were injected with 200 ng pertussis toxin in PBS on day 0 and 2 days after transfer.

### T cell purification and differentiation.

Naive CD4^+^ T cells (CD4^+^CD44^lo^CD62L^hi^CD25^–^) were purified by flow cytometry. IL-6, TGF-β, and IL-6/IL-6R protein chimeras were purchased from R&D Systems. IL-23 and IL-1β were purchased from PeproTech. Naive T cells were stimulated with plate-bound anti-CD3 (2 μg/mL) and anti-CD28 (2 μg/mL) antibodies in the presence of anti–IL-4 (10 μg/ml; 11B11; BioLegend) and anti–IFN-γ (10 μg/ml; XMG1.2; BioLegend) to generate Th0 cells; IL-6 (30 ng/ml), TGF-β1 (2 ng/ml), anti–IL-4, and anti–IFN-γ antibodies were added to induce Th17 cell differentiation. Pathogenic Th17 cells were generated in the presence of IL-6 (20 ng/ml), IL-1β (20 ng/ml), IL-23 (20 ng/ml), anti–IL-4, and anti–IFN-γ. Tregs were generated in the presence of IL-2 (10 ng/mL) and TGF-β1 (5 ng/mL). For the induction of Th1 cells, 25 ng/ml rmIL-12 and 10 μg/ml anti–IL-4 were used. Regarding Th2 cells, 10 ng/mL mIL-4 and 10 μg/mL anti–IFN-γ were applied. At the end of the culture period of different Th differentiation conditions, we restimulated CD4^+^ T cells with PMA (50 ng/mL) and ionomycin (500 ng/mL) in the presence of 2 μM monensin for 4 hours for intracellular staining or only restimulated with PMA and ionomycin for 4 hours for the quantification of mRNA expression.

### In vitro Treg suppression assay.

Effector T cells (CD4^+^CD25^–^) were obtained from WT mice by magnetic separation and subsequently labeled with 5 nM CFSE (Invitrogen) for 10 minutes at 37°C. CFSE-labeled effector T cells (1 × 10^5^) were then cocultured with Tregs (CD4^+^CD25^+^) according to the indicated ratios in the presence of anti-CD3 (clone: 145-2C11;BD) and anti-CD28 (clone: 37.51; BD). After 72 hours, the cells were harvested to measure CFSE dilution by flow cytometry.

### Flow cytometry and related reagents.

At scarify, single-cell suspensions were isolated from dLNs, spleens, and CNS. Briefly, the brain and spinal cord were obtained, homogenized, and then incubated with collagenase D (2.5 mg/mL, Roche Diagnostics) and DNase I (1 mg/mL, MilliporeSigma) for 30 minutes. Mononuclear cells were enriched by gradient centrifugation at 670*g* for 30 minutes on a 37%/70% Percoll gradient without interruption. Before staining, cells were blocked with anti-CD16/CD32 antibodies. The following antibodies were used for flow cytometry: anti-CD3 (OKT3), anti-CD45 (30-F11), anti-MHCII (M5/114.15.2), anti-CD8 (53-6.7), anti-CD44 (IM7), anti-Foxp3(FJK-16S), anti–GM-CSF (MP1-22E9), anti-CD49d (R1-2), anti–IL-17A (eBio17B7), and anti–IFN-γ (XMG1.2) (eBioscience); anti-CD4 (GK1.5), anti-CD25 (PC61.5), anti-CD62L (MEL-14), anti–Ly-6G/Gr-1 (1A8-Ly6g),anti-F4/80 (BM8), anti-CCR2 (SA203G11), anti-CCR6 (29-2L17), and anti-CD29 (HMβ1-1) (BioLegend). In addition, anti–p-STAT3 (Y705) (catalog 557814) and p-STAT1 (Y701) (catalog 502069) antibodies were purchased from BD Biosciences. Phosphoflow cytometry analysis was performed using BD Phosflow buffers (554655 and 558050). Stained cells were analyzed by FACSCanto flow cytometer and were analyzed with FlowJo software (Tree Star).

### Quantitative RT-PCR.

Total RNA samples were extracted with TRIzol reagent and then reverse-transcribed to cDNA according to the manufacturer’s instructions. Quantitative real-time PCR (RT-PCR) was performed using SYBR Green (Thermo Fisher Scientific) with a Roche LightCycler 480 system. All the primers used for real-time PCR are listed in Supplemental Table 1. The condition for real-time PCR was 40 cycles at 94°C for 15 seconds followed by 40 cycles at 60°C for 60 seconds.

### Western blot analysis.

Cell lysates were prepared in RIPA buffer containing protease inhibitor cocktail and phosphatase inhibitor cocktail. Proteins were subjected to PAGE gels and transferred to nitrocellulose membranes and subsequently probed with anti–p-STAT3 (Cell Signaling Technology, catalog 9145, 1:1,000 dilution), anti-STAT3 (Cell Signaling Technology, catalog 4904,1:1,000), anti–p-JAK2 (Cell Signaling Technology, catalog 3771, 1:1,000 dilution), anti–p-ERK (Cell Signaling Technology, catalog 4370, 1:1,000 dilution), anti-ERK(Cell Signaling Technology, catalog 4695, 1:1,000 dilution), anti–p-Akt (Cell Signaling Technology, catalog 4060, 1:1,000 dilution), anti-Akt (Cell Signaling Technology, catalog 4691, 1:1,000 dilution), anti–IL-6R (Santa Cruz Biotechnology Inc., catalog sc373708, 1:1,000 dilution), anti–IL-6 (Cell Signaling Technology, catalog 12153, 1:1,000 dilution), anti-FLAG (Sigma-Aldrich; catalog F3165, 1:1,000 dilution) and anti–c-Myc (Sigma-Aldrich, catalog C3956, 1:1,000 dilution). The membranes were then incubated with appropriate secondary antibodies and developed with Amersham ECL (GE Healthcare).

### Immunoprecipitation.

HEK293T cells and Jurkat cells purchased from ATCC were cultured according to the manufacturer’s instructions. Immunoprecipitation was performed as we previously described (34). Briefly, various cells were lysed in a lysis buffer (20 mM Tris-HCl, pH 7.5; 150 mM NaCl; 0.5 mM EDTA; 1.5 mM MgCl_2_; 0.1%NP-40; 10% glycerol and protease inhibitor cocktail) at 4°C for 30 minutes. Integral membrane protein utilized a 2-phase partitioning system to efficiently separate (Thermo Mem-PER Plus, catalog 89842). Equal amounts of protein were immunoprecipitated with anti–IL-6R or anti-CTRP4 antibodies that were homemade, as described in a previous study (35), and bound to Protein G Sepharose (GE Healthcare) or anti-FLAG M2 Affinity Gel (catalog A2220, MilliporeSigma). After extensive washing 5 times with lysis buffer, the indicated proteins were subjected to Western blot analysis.

### Radio-ligand binding assay.

rhCTRP4 protein was purified from CTRP4-CHO cells as described (13). CTRP4 was labeled with ^125^I in 0.01 N PB buffer with chloramine T at 4°C for 30 seconds, followed by elution with 0.01 N PB buffer in a SEPHADEX-G25 column. For saturation experiments, the equivalent quantity of Jurkat cell membrane extract was incubated with different 2-fold serial dilutions of [^125^I]-CTRP4. Nonspecific binding was measured in the presence of a 500-fold excess of CTRP4 at each concentration of [^125^I]-CTRP4. For competition-binding assays, the equivalent quantities of cell-membrane extract and [^125^I]-CTRP4 were incubated with different concentrations of unlabeled CTRP4, OSM, or IL-6. The reaction was incubated for 24 hours at 4°C and 25% PEG was added, followed by counting on a γ counter.

### ELISA.

Human IL-6R (R&D) was precoated onto plates and maintained overnight at 4°C and then incubated with human IL-6 or rhCTRP4. The plates were blocked with 200 μl of 1% BSA in PBS for 2 hours, followed by incubating with anti–IL-6R primary antibody. The color changes were read at OD 450. Under different Th17 differentiation conditions, supernatants from cell cultures were collected and measured for cytokines secreted by IL-17A, and the IFN-γ ELISA Kit was used (R&D SM1700; MIF00). The levels of MOG-specific antibodies and the levels of CTRP4 were determined by the Anti-Mouse MOG Antibody Quantitative ELISA Kit (Anaspec) and the Mouse Complement C1q Tumor Necrosis Factor–Related Protein 4 ELISA Kit (Abbexa) according to the manufacturer’s instructions.

### Histological assay of spinal cord sections.

The spinal cords were dissected and fixed with 4% PFA, dehydrated, and embedded in paraffin. For immunofluorescence staining, sections were incubated at 55°C for 30 minutes for antigen retrieval. The sections were pretreated with a 0.3% solution of H_2_O_2_ to block endogenous peroxidase activity. The sections were then incubated with 10% goat serum in PBS-T, followed by incubating with the primary antibodies, including anti–IL-17A (Invitrogen, 14-7179-80, 1:100 dilution) and anti-CD4 (Invitrogen, 14-9766-82, 1:100 dilution). Next, the sections were detected with Alexa Fluor 555–labeled streptavidin at room temperature for 1 hour. After staining with DAPI to visualize cell nuclei, the slides were analyzed by fluorescence microscopy.

### Lentiviral infection.

The IL-6R, gp130, or CTRP4 cDNA was subcloned into lentivirus vector TG006. For lentivirus package and production, HEK293T cells were cotransfected with 10 μg of TG006-gp130 transfer vector and 5 μg of psPAX2 and 5 μg of VSVG packing vector in 1.5 mL of Opti-MEM used with Lipofectamine 2000 reagent. Seventy-two hours after transfection, lentivirus particles were harvested, filtered, and added to Ba/F3 cells or Jurkat in the presence of polybrene (8 μg/ml). After 24 hours, the medium containing the viral particles was replaced by the viral particle–free culture medium. The cells were cultured and maintained in culture medium containing 2 μg/ml puromycin to obtain target cells.

### Bone marrow chimeras.

The recipient mice were exposed to lethal-dose γ irradiation (10 Gy) to destroy hematopoietic stem cells. After a 2-hour recovery period, bone marrow cells derived from the tibiae and femurs of donor mice aged between 2 and 4 months were intravenously injected into irradiated recipients (5 × 10^6^/mouse). The chimeric mice were housed for a total of 8 weeks for the complete recovery of the hematopoietic niche and then subjected to EAE induction as described previously.

### Statistics.

Our data were randomly collected. Experimental results were analyzed for significance using Student’s *t* test and Mann-Whitney *U* test for 2 groups. Statistical significance also was assessed by 1-way ANOVA or 2-way ANOVA followed by Bonferroni’s or Dunnett’s multiple-comparisons test for 3 or more groups. Statistical analyses were performed using GraphPad Prism (version 9.0).

### Study approval.

Animal studies were conducted according to the guidelines of the Institutional Animal Care and Use Committee of Peking University under the approval of the Ethical Office of Peking University People’s Hospital (project license 2022PHE107).

### Data availability.

Values associated with the main manuscript and supplemental material, including values for all data points shown in the graphs and values behind any reported means, are reported in the Supporting Data Values file.

## Author contributions

LC designed and performed experiments, analyzed results, and wrote the manuscript. WC, JD, MH, and NZ conducted the experiments and analyzed data. HH and LL collected and analyzed data. QL, XZ, and LW supervised the study and writing of the manuscript.

## Supplementary Material

Supplemental data

Supporting data values

## Version 1 11/28/2023

In-Press Preview

## Version 2 02/15/2024

Electronic publication

## References

[1] Goverman J (2009). Autoimmune T cell responses in the central nervous system. Nat Rev Immunol.

[2] Korn T (2017). T cell responses in the central nervous system. Nat Rev Immunol.

[3] Moser T (2020). The role of TH17 cells in multiple sclerosis: Therapeutic implications. Autoimmun Rev.

[4] Miossec P (2012). Targeting IL-17 and TH17 cells in chronic inflammation. Nat Rev Drug Discov.

[5] Bettelli E (2006). Reciprocal developmental pathways for the generation of pathogenic effector TH17 and regulatory T cells. Nature.

[6] Kebir H (2007). Human TH17 lymphocytes promote blood-brain barrier disruption and central nervous system inflammation. Nat Med.

[7] Harbour SA-O (2020). T_H_17 cells require ongoing classic IL-6 receptor signaling to retain transcriptional and functional identity. Sci Immunol.

[8] Ciofani M (2012). A validated regulatory network for Th17 cell specification. Cell.

[9] Luo Y (2016). Expression of the novel adipokine C1qTNF-related protein 4 (CTRP4) suppresses colitis and colitis-associated colorectal cancer in mice. Cell Mol Immunol.

[10] Li Y (2020). C1q/TNF-related protein 4 induces signal transducer and activator of transcription 3 pathway and modulates food intake. Neuroscience.

[11] Ye L (2021). C1q/TNF-related protein 4 restores leptin sensitivity by downregulating NF-κB signaling and microglial activation. J Neuroinflammation.

[12] Byerly MS (2014). C1q/TNF-related protein 4 (CTRP4) is a unique secreted protein with two tandem C1q domains that functions in the hypothalamus to modulate food intake and body weight. J Biol Chem.

[13] Cao L (2021). CTRP4 acts as an anti-inflammatory factor in macrophages and protects against endotoxic shock. Eur J Immunol.

[14] Pullabhatla V (2018). De novo mutations implicate novel genes in systemic lupus erythematosus. Hum Mol Genet.

[15] Vester SK (2021). Nucleolin acts as the receptor for C1QTNF4 and supports C1QTNF4-mediated innate immunity modulation. J Biol Chem.

[16] Reboldi A (2009). C-C chemokine receptor 6-regulated entry of TH-17 cells into the CNS through the choroid plexus is required for the initiation of EAE. Nat Immunol.

[17] Lacroix M (2015). Novel insights into interleukin 6 (IL-6) cis- and trans-signaling pathways by differentially manipulating the assembly of the il-6 signaling complex. J Biol Chem.

[18] Durant L (2010). Diverse targets of the transcription factor STAT3 contribute to T cell pathogenicity and homeostasis. Immunity.

[19] Houston S (2023). STAT3 and autoimmunity. Nat Immunol.

[20] Quintana FJ (2017). Old dog, new tricks: IL-6 cluster signaling promotes pathogenic T_H_17 cell differentiation. Nat Immunol.

[21] Baran P (2013). Minimal interleukin 6 (IL-6) receptor stalk composition for IL-6 receptor shedding and IL-6 classic signaling. J Biol Chem.

[22] Siddiquee K (2007). Selective chemical probe inhibitor of Stat3, identified through structure-based virtual screening, induces antitumor activity. Proc Natl Acad Sci U S A.

[23] Tanaka S (2014). Sox5 and c-Maf cooperatively induce Th17 cell differentiation via RORγt induction as downstream targets of Stat3. J Exp Med.

[24] Poholek CH (2020). Noncanonical STAT3 activity sustains pathogenic Th17 proliferation and cytokine response to antigen. J Exp Med.

[25] Whitley SA-O (2022). Local IL-23 is required for proliferation and retention of skin-resident memory T_H_17 cells. Sci Immunol.

[26] Mufazalov IA (2020). Cutting Edge: IL-6-driven immune dysregulation is strictly dependent on IL-6R α-chain expression. J Immunol.

[27] Zhang C (2016). CD5 Binds to interleukin-6 and induces a feed-forward loop with the transcription factor STAT3 in B Cells to Promote Cancer. Immunity.

[28] Soutto M (2019). Activation of STAT3 signaling is mediated by TFF1 silencing in gastric neoplasia. Nat Commun.

[29] Nagashima H (2014). The adaptor TRAF5 limits the differentiation of inflammatory CD4(+) T cells by antagonizing signaling via the receptor for IL-6. Nat Immunol.

[30] Freyer CW (2020). Cytokine release syndrome and neurotoxicity following CAR T-cell therapy for hematologic malignancies. J Allergy Clin Immunol.

[31] Luo W (2020). Targeting JAK-STAT signaling to control cytokine release syndrome in COVID-19. Trends Pharmacol Sci.

[32] Yousif AS (2021). The persistence of interleukin-6 is regulated by a blood buffer system derived from dendritic cells. Immunity.

[33] Saligrama N (2019). Opposing T cell responses in experimental autoimmune encephalomyelitis. Nature.

[34] Feng H (2014). EGFR phosphorylation of DCBLD2 recruits TRAF6 and stimulates AKT-promoted tumorigenesis. J Clin Invest.

[35] Huang H (2016). Preparation and identification of monoclonal antibody against C1q/TNF-related protein 4. Monoclon Antib Immunodiagn Immunother.

