# Supplementary material for: CTRP4/interleukin-6 receptor signaling ameliorates autoimmune encephalomyelitis by suppressing Th17 cell differentiation
Source: J Clin Invest. 2023 Nov 28;134(4):e168384. doi: 10.1172/JCI168384 (PMC10866667; doi:10.1172/JCI168384)
Supplement: Supplemental data [file jci-134-168384-s205.pdf]

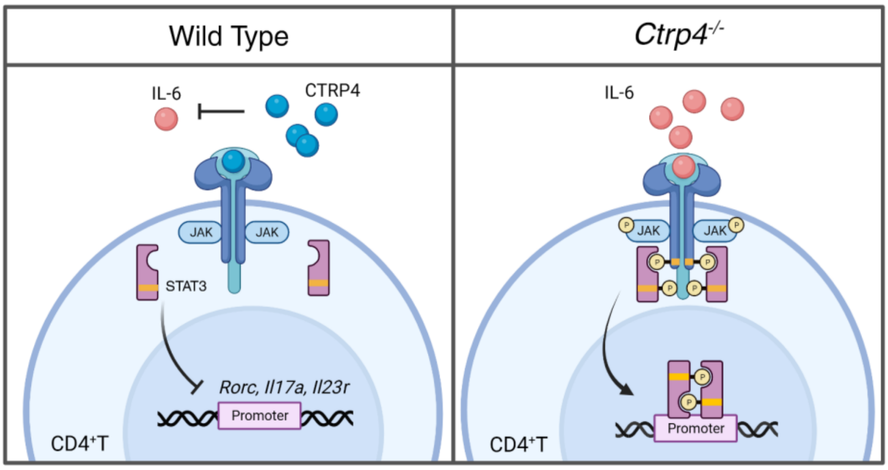

Graphical Abstract

Supplemental Figure

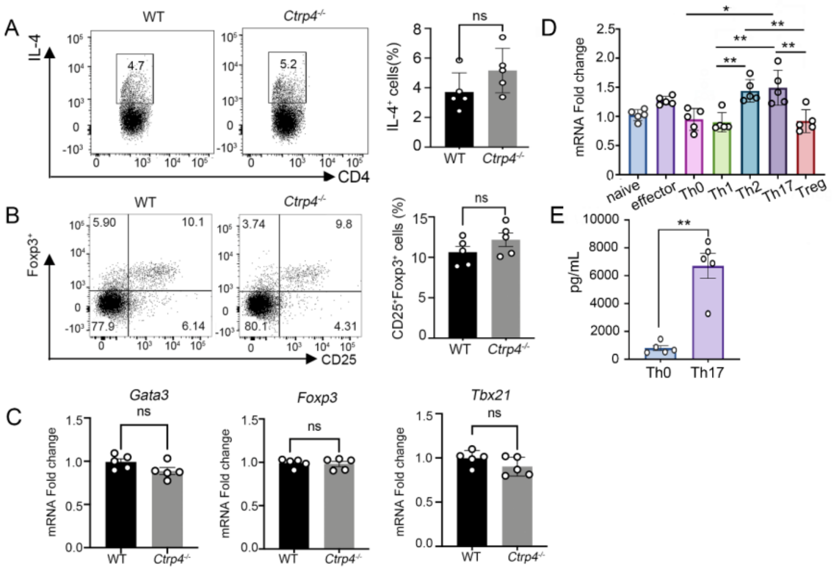

Supplementary Figure1: Effect of CTRP4 on T cell subsets

(A) Flow cytometry analyses of Th2 effector T cells ($CD4^+CD44^+$ $IL\text{-}4^+$ cells) in the spleen of *Ctrp4*$^{-/-}$ and WT mice.

(B) Flow cytometry analysis of Treg cells ($CD4^+CD25^+Foxp3^+$) in the spleen of *Ctrp4*$^{-/-}$ and WT mice.

(C) Gene expression levels of *Gata3, Foxp3* or *Tbx21* in $CD4^+$ T cells were analyzed by quantitative real-time PCR.

(D) Gene expression of *Ctrp4* was shown as summary bar graph. Naive $CD4^+CD62L^{high}CD44^{low}CD25^-$ T cells were sorted from WT mice were stimulated with anti-CD3 and anti-CD28 antibodies to obtain effector $CD4^+$ T cells, or differentiated towards Th subsets under Th0/1/2/17 or Treg differentiation condition. Real-time RT-PCR of *Ctrp4* mRNA expression was analyzed and normalized against *Gapdh*.

(E) The productions of CTRP4 of WT $CD4^+$T cells under Th0 or Th17 differentiation conditions were quantitated by ELISA, respectively. Data were shown as mean ± SEM and were from one of three

independent experiments with similar results. one-way ANOVA with Tukey's post-test was used for **D**. Statistical significance was determined using unpaired Student t test or Mann-Whitney U test for **A-C** and **E**; $^{**}p < 0.01$, $^{***}p < 0.001$, ns not significance.

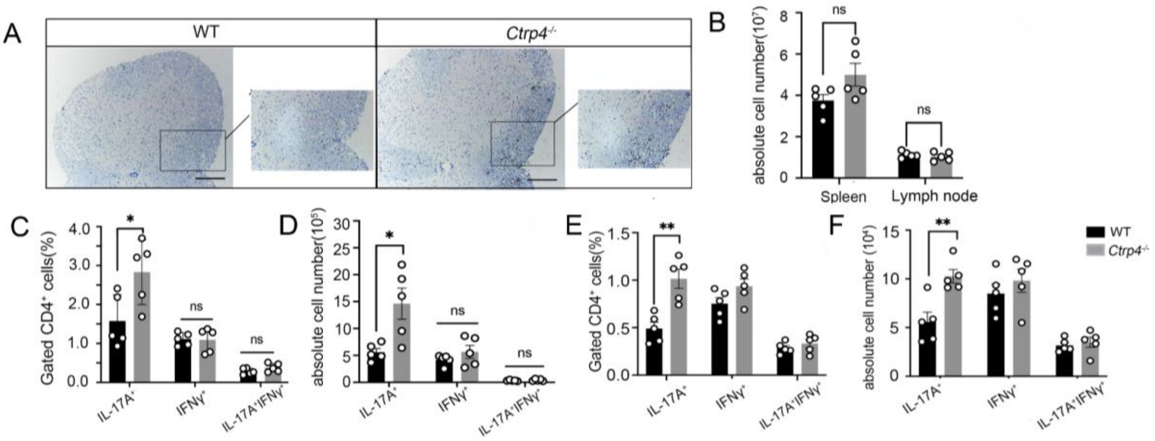

Supplementary Figure2: *Ctrp4* deficiency exacerbates EAE progression with increased infiltration of CD4$^+$IL-17A$^+$T cells in the peripheral.

(A) Representative immunohistochemistry images of CD4 expression in the spinal cord of indicated EAE animals at the peak of disease were performed. Scale bar,100 µm.

(B) The summary bar graph showed the absolute number of CD4$^+$ in spleens and draining lymph nodes of *Ctrp4$^{-/-}$* and WT mice at the peak of disease.

(C-D) Flow-cytometric analysis of the frequencies **(C)** or the absolute cell numbers **(D)** or of CD4$^+$IL-17$^+$, CD4$^+$IL-17$^+$IFNγ$^+$, CD4$^+$IFNγ$^+$ cells in the spleens harvested from WT and *Ctrp4$^{-/-}$* mice at day18 postimmunization.

(E-F) Flow-cytometric analysis of the frequencies **(E)** or the absolute cell numbers **(F)** of CD4$^+$IL-17$^+$, CD4$^+$IL-17$^+$IFNγ$^+$, CD4$^+$IFNγ$^+$ cells in the draining lymph nodes isolated from WT and *Ctrp4$^{-/-}$* mice at day18 postimmunization. Data were shown as mean ± SEM and were from one of three independent experiments with similar results. Statistical significance was determined using unpaired Student t test or Mann-Whitney U test; $^{*}p < 0.05$; $^{**}p < 0.01$, ns not significance.

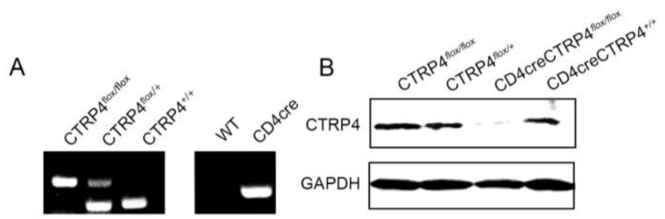

Supplementary Figure3: The deletion efficacy of T cell condition CTRP4 KO mice

(A) Genotyping by PCR analysis of CTRP4$^{flox/flox}$, CTRP4$^{+/+}$ and heterozygous mice or PCR analysis of CD4 Cre transgene mice with primers designed for indicated sites.

(B) CD4$^+$T cells from CTRP4$^{flox/flox}$, CTRP4$^{+/flox}$, CD4creCTRP4$^{flox/flox}$, or CD4creCTRP4$^{+/+}$ mice were purified and lysates were subjected to western blot analysis for CTRP4 protein expression.

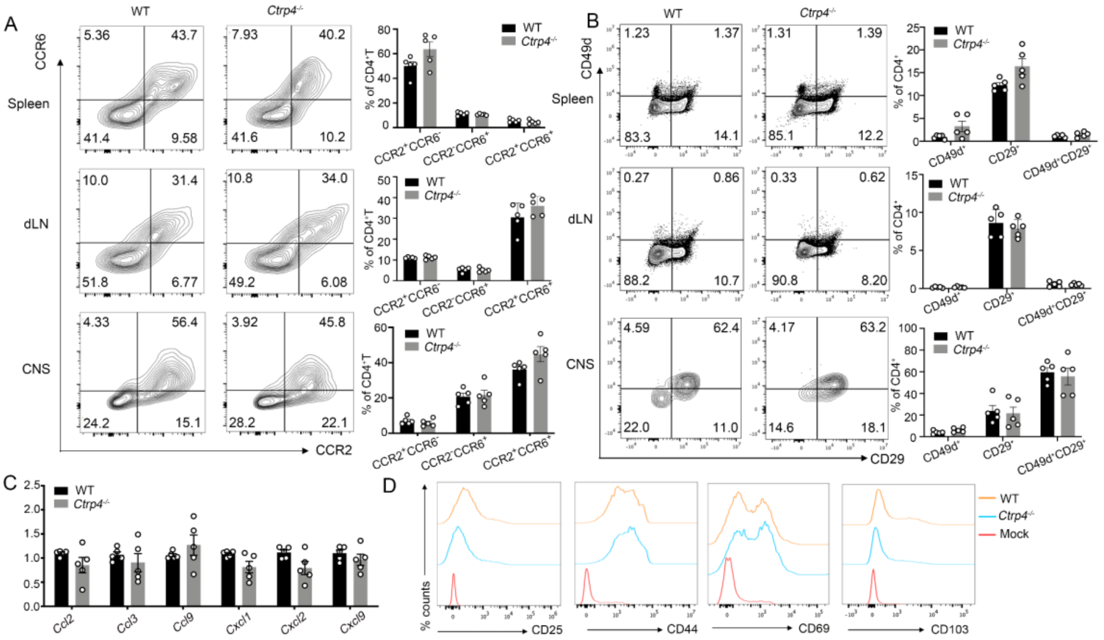

Supplementary Figure 4: CTRP4 deletion did not affect the ability of Th17 cells to activate or migrate into CNS

(A) Representative flow cytometric analysis and quantification of CCR6/CCR2 expression in $CD4^+$T cells from *Ctrp4*$^{-/-}$ and WT in the dLNs, spleens or CNS on day18 post EAE induction.

(B) Representative flow cytometric analysis and quantification of CD49d and CD29 expression in $CD4^+$ T cells from spleens, lymph nodes and CNS at peak stage of disease.

(C) Quantitative PCR analysis to determine the expression levels of indicated genes encoding multiple chemokines in spinal cord of $MOG_{35\text{-}55}$-immunized *Ctrp4*$^{-/-}$ and WT at the peak of disease.

(D) Comparable activation status of $CD4^+$ T cells between *Ctrp4*$^{-/-}$ and WT mice on day18 post EAE induction were analyzed by flow cytometry. Data were shown as mean ± SEM and were from one of three independent experiments with similar results. Statistical significance was determined using unpaired Student t-test or Mann-Whitney U test.

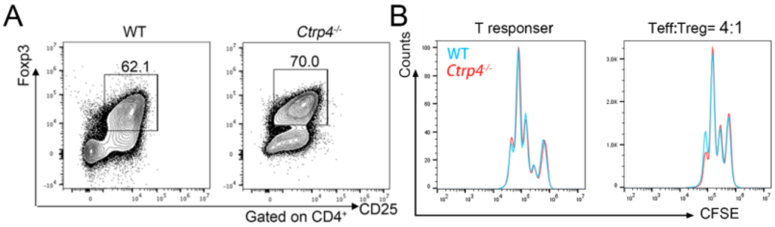

Supplementary Figure 5: The in vitro differentiation ability of naïve $CD4^+$ T cells into Treg cells was not impaired in CTRP4 KO mice

(A) Naïve $CD4^+CD62L^{high}CD44^{low}CD25^-$ T cells were sorted from WT and Ctrp4$^{-/-}$ mice, and differentiated with 5 ng/ml TGFβ1 and 5 ng/ml IL-2 for 5 days. Numbers adjacent to outlined areas indicated the percentage of $CD4^+CD25^+Foxp3^+$ cells.

(B) CFSE-labeled effector $CD4^+$ T cells were cocultured with WT and *Ctrp4*$^{-/-}$Treg cells to conduct the Treg suppression assay. The suppression capacity was determined through CFSE dilution when effector T cells cultured at a 4:1 ratio with Treg cells. Data were from one of three independent experiments with similar results.

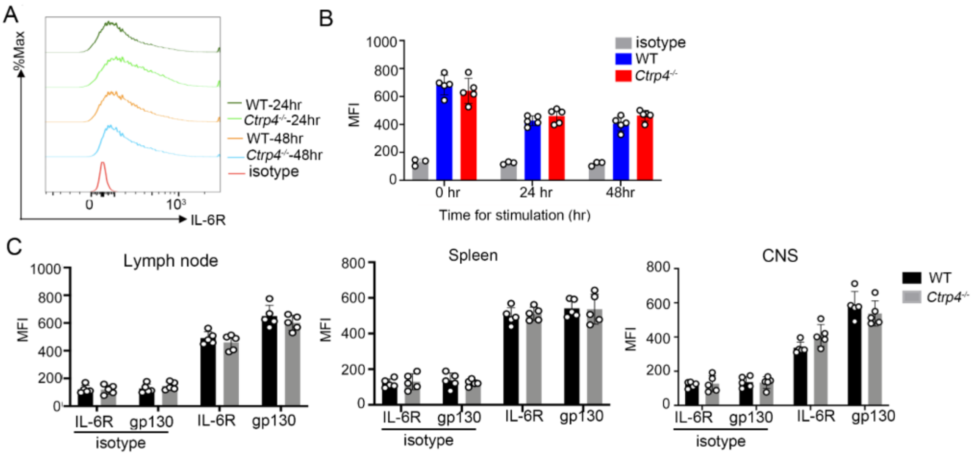

Supplementary Figure 6: Ctrp4 deficiency did not alter the expression level of IL-6R

(A) The representative histograms showed the expression level of IL-6R on purified wild-type and *Ctrp4*$^{-/-}$ naïve CD4^{+}T cells stimulated with anti-CD3 and anti-CD28 for indicated time.

(B) Flow cytometric analysis of the mean fluorescence intensity (MEI) of IL-6R on purified wild-type and *Ctrp4*$^{-/-}$ naïve CD4^{+}T cells stimulated with anti-CD3 and anti-CD28 for indicated time. Isotype means isotype-matched control antibody.

(C) Flow cytometric analysis of the mean fluorescence intensity (MEI) of gp130 or IL-6R on CD4^{+} T cells isolated from CNS and peripheral lymphoid organs of *Ctrp4*$^{-/-}$ and WT mice on day18 post EAE induction. Data were shown as mean ± SEM and were from one of three independent experiments with similar results. Statistical significance was determined using unpaired Student t-test or Mann-Whitney U test.

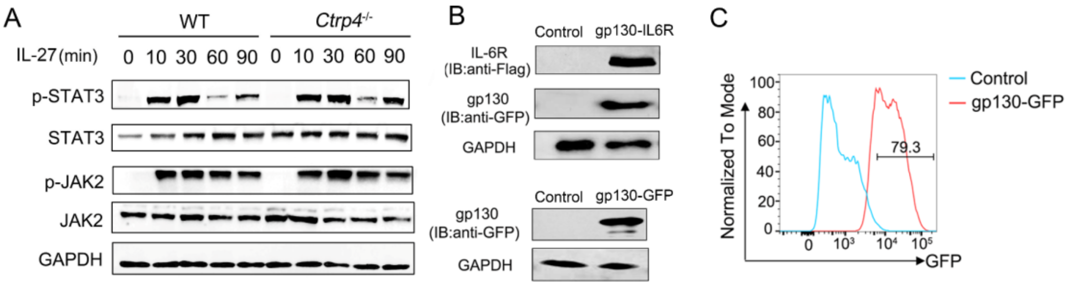

Supplementary Figure 7: CTRP4 responded to IL-6 rather than other gp130 family cytokines

(A) Purified CD4^{+}T cells from *Ctrp4*$^{-/-}$ and WT mice were stimulated with IL-27 (50 ng/mL) for indicated time. Lysates were subjected to western blot analysis for phosphorylated JAK2, p-JAK2, p-STAT3, STAT3 and GAPDH (as a control). The samples derived from the same experiment and that gels/blots were processed in parallel.

(B) The expression levels of gp130 and IL-6R in Ba/F3-gp130-IL-6R cells lysates were analyzed by indicated antibodies (up). The expression level of gp130 in Ba/F3-gp130 cells lysates was analyzed by anti-GFP antibodies (bottom).

(C) Flow cytometric analysis of the expression of gp130 in Ba/F3-gp130 cells. Data were from one of three independent experiments with similar results.

Supplementary Table1: Primer sequence used for qPCR assays.

Gene	Forward Primer	Reverse Primer
Il17a	CTCCAGAAGGCCCTCAGACTC	GGGTCTTCATTGCGGTGG
Ifng	TCGAATCGCACCTGATCACTA	GGGTTGTTCACCTCGAACTTG
Rorc	CCGCTGAGAGGGCTTCAC	TGCAGGAGTAGGCCACATTAC
Il17f	CCCCATGGGATTACAACATCC	CATTGATGCAGCCTGAGTGTT
II23r	AACATGACATGCACCTGGAA	TCCATGCCTAGGGAATTGAC
Foxp3	CCCATCCCCAGGAGTCTTG	ACCATGACTAGGGGCACTGTA
Tbx21	GCCAGGGAACCGGTTATATG	GACGATCATCTGGGTCACAT
Gata3	AAGCTCAGTATCCGCTGACG	GTTTCCGTAGTAGGACGGGAC
II6ra	CATTGCCATTGTTCTGAGGTTC	AGTAGTCTGTATTGCTGATGTC
Gapdh	GACTTCAACAGCAACTCCCAC	TCCACCACCCTGTTGCTGTA
Ccl2	CCGGCTGGAGCATCCACGTGT	TGGGGTCAGCACAGACCTCTCTCT
Ccl20	CGACTGTTGCCTCTCGTACA	GAGGAGGTTCACAGCCCTTT
Cxcl1	CACAGGGGCGC CTATCGCCAA	CAAGGCAAGCCTCGCGACCAT
Cxcl2	ACCCCACTGCGCCCAGACAGAA	AGCAGCCCAGGCTCCTCCTTTCC
Ccl3	TGTACCATGACACTCTGCAAC	CAACGATGAATTGGCGTGGAA
Cx3cl1	ACGAAATGCGAAATCATG TGC	CTGTGTCGTCTCCAGGACAA
Ccl9	CCCTCTCCTTCCTCATTCTTACA	AGTCTTGAAAGCCCATGTGAAA
Cxcl9	TCCTTTTGGGCATCATCTTCC	TTTGTAGTGGATCGTGCCTCG
Cxcl11	GGCTTCCTTATGTTCAAACAGGG	GCCGTTACTCGGGTAAATTACA
